# Structural and functional analyses of a germline *KRAS* T50I mutation provide insights into Raf activation

**DOI:** 10.1172/jci.insight.168445

**Published:** 2023-09-08

**Authors:** Pan-Yu Chen, Benjamin J. Huang, Max Harris, Christopher Boone, Weijie Wang, Heidi Carias, Brian Mesiona, Daniela Mavrici, Amanda C. Kohler, Gideon Bollag, Chao Zhang, Ying Zhang, Kevin Shannon

**Affiliations:** 1 Department of Pediatrics, UCSF, San Francisco, California, USA.; 2 Plexxikon Inc., South San Francisco, California, USA.; 3 Helen Diller Family Comprehensive Cancer Center, UCSF, San Francisco, California, USA.

**Keywords:** Oncology, Leukemias, Oncogenes

## Abstract

A T50I substitution in the K-Ras interswitch domain causes Noonan syndrome and emerged as a third-site mutation that restored the in vivo transforming activity and constitutive MAPK pathway activation by an attenuated *Kras^G12D,E37G^* oncogene in a mouse leukemia model. Biochemical and crystallographic data suggested that K-Ras^T50I^ increases MAPK signal output through a non-GTPase mechanism, potentially by promoting asymmetric Ras:Ras interactions between T50 and E162. We generated a “switchable” system in which K-Ras mutant proteins expressed at physiologic levels supplant the fms like tyrosine kinase 3 (FLT3) dependency of MOLM-13 leukemia cells lacking endogenous *KRAS* and used this system to interrogate single or compound G12D, T50I, D154Q, and E162L mutations. These studies support a key role for the asymmetric lateral assembly of K-Ras in a plasma membrane–distal orientation that promotes the formation of active Ras:Raf complexes in a membrane-proximal conformation. Disease-causing mutations such as T50I are a valuable starting point for illuminating normal Ras function, elucidating mechanisms of disease, and identifying potential therapeutic opportunities for Rasopathy disorders and cancer.

## Introduction

Ras proteins are small GTPases that cycle between active GTP-bound and inactive GDP-bound states to regulate diverse cellular processes. In cancer cells, highly prevalent somatic mutations that alter amino acids 12, 13, and 61 impair intrinsic GTP hydrolysis, induce resistance to GAP stimulation, and/or increase the rate of nucleotide exchange. Consequently, these mutant Ras proteins accumulate in the GTP-bound state, constitutively increase effector pathway signaling, and drive aberrant cell proliferation and survival. The development of K-Ras^G12C^ inhibitors with antitumor activity has demonstrated the feasibility of targeting these previously “undruggable” oncoproteins and showed that *KRAS*-mutant cancers remain addicted to hyperactive Ras in vivo (1–3). Studies of tumor specimens obtained at disease progression indicate selective pressure to restore oncogenic Ras signaling through both on- and off-target adaptive resistance mechanisms (4, 5). These observations are consistent with previous work that emphasized the importance of developing effective strategies to both potently inhibit mutant Ras signal output and target possible bypass mechanisms (6, 7).

A distinct class of germline *KRAS* and *NRAS* mutations causes Rasopathy developmental disorders (8–10). These alleles encode gain-of-function K-Ras proteins that are biochemically and functionally attenuated in comparison with Ras oncoproteins, allowing compatibility with embryonic development (8). Strikingly, some of these mutant proteins harbor substitutions in amino acids distant from Ras effector or nucleotide binding sites and exhibit normal intrinsic GTPase activity, responsiveness to GAPs, and nucleotide exchange (9–11). Interestingly, approximately 40% of Rasopathy *BRAF* mutations alter amino acids predicted to mediate interactions between Ras and the cysteine-rich domain (CRD) of B-Raf (12).

Prior to interacting with Ras and being recruited to the plasma membrane (PM), Raf is held in an autoinhibited state by 14-3-3 scaffold proteins and MEK (13). The Raf-CRD occupies the center position of this complex. Recent functional studies showed that many Rasopathy B-Raf mutant proteins are characterized by impaired kinase autoinhibition and that this correlates with their phenotypic severity (14).

In the absence of effector engagement, Ras-GTP likely exists in a membrane-distal state, which increases its interaction range to recruit Raf to the PM (15). Subsequent K-Ras binding to the exposed surfaces of Raf releases CRD-mediated autoinhibitory interactions and integrates kinase domain–exposed Raf into a membrane-proximal signaling conformation (13, 16). Recently solved co-crystal structures of active Ras in complex with Raf conserved region 1 (17, 18) confirmed earlier predictions that efficient Raf activation by Ras requires both a high-affinity interaction with the Ras binding domain (RBD) of Raf and low-affinity binding to the CRD (19, 20). In the Ras signalosome, both Ras and the Raf-CRD are involved in membrane lipid binding based on NMR-guided characterization of nanodisc-bound Ras:Raf complexes (21).

A germline *NRAS* mutation encoding a threonine-to-isoleucine substitution at codon 50 (T50I) causes Rasopathy disorders (10). Somatic *KRAS^T50I^* mutations were subsequently identified in a few melanomas and colorectal cancers (22, 23). An unrelated line of investigation converged on T50I when a *Kras^G12D^* oncogene impaired for Raf binding due to a second-site E37G substitution initiated T lineage acute lymphoblastic leukemia in a murine retroviral transduction and transplantation system (24). Two of these leukemias independently acquired T50I as a third-site mutation that restored the oncogenic activity of *Kras^G12D,E37G^*. Taken together, these diverse observations in different disease contexts support an unexpected role of T50 in regulating Ras activity. T50 is located on the b2–b3 loop of Ras between the effector-binding switch 1 and 2 regions and was predicted to activate signal output through a non-GTPase mechanism, based on biochemical and structural studies and in silico modeling (10, 24).

Here, we describe comprehensive analyses of mutant K-Ras4b proteins (hereafter referred to as K-Ras) containing T50I as a single mutation and in combination with substitutions at other residues implicated in Ras:Ras lateral assembly. We solved the crystal structure of K-Ras^T50I^ bound to GDP and generated a disease-relevant isogenic cell line model system for functional and biochemical studies wherein various K-Ras proteins were expressed at physiologic levels in cells lacking endogenous K-Ras. Integrating our results with recently published models of Ras:Raf activation based on co-crystal structures and functional studies (13, 17, 18), we propose that T50I enhances Ras signal output by promoting asymmetric lateral assembly of individual Ras molecules at the PM and by possibly enhancing the interaction of Ras with the Raf-CRD. Together, this work underscores the value of disease-relevant mutations as a starting point for interrogating mechanistic questions. We further describe a versatile approach for generating isogenic models for interrogating Ras function and testing the specificity and selectivity of candidate therapeutics that can be applied to other cancers characterized by druggable driver kinase mutations.

## Results

Our previous in silico analysis suggested that T50I might promote Ras/MAPK activation by bringing together the G domains of neighboring Ras molecules in an asymmetric manner (24). In this model, the interswitch b2–b3 loop–mediated asymmetric protein–protein interface allows flexible, higher order assembly via head to tail juxtaposition (Supplemental Figure 1A; supplemental material available online with this article; https://doi.org/10.1172/jci.insight.168445DS1). This contrasts with symmetric dimer models proposed by others from analyses of subunit packing in published structures of Ras (25–28). Cirstea et al. solved the crystal structure of H-Ras^T50I^ bound to GTP (10) and performed molecular dynamic simulations that implicated the Ras b2–b3 loop and α5 helix as a cryptic third-switch region that undergoes a conformational change upon GTP binding (switch III) (29) (Supplemental Figure 1B). On the basis of these data, they speculated that T50I enhances MAPK signaling by reorienting the G domains of Ras monomers relative to the PM (10).

To gain additional insights into how T50I might increase Ras signal output and overcome the deleterious effects of an E37G mutation, we first reviewed the K-Ras^G12D^ structure (Protein Data Bank [PDB]: 5USJ) in the context of recent co-crystal structures of K-Ras in complex with the RBD of Raf (PDB: 4G0N; 6VJJ) or with the RBD and CRD (PDB: 6XI7; 7JHP). The observation that E37 of Ras forms cross β-strand salt bridges with R59 and R67 of Raf (30) (Supplemental Figure 1C) provides a molecular explanation for the decreased Raf binding, reduced transforming activity, and impaired MAPK pathway activation of K-Ras^G12D,E37G^ (24, 31). Because T50 is located more than 25 Å away from E/G37 (Supplemental Figure 1C), a large-scale structural change must occur for the T50I mutation to directly compensate for the loss of cross–β-strand interactions due to the E37G substitution. We formally tested this possibility by solving the crystal structure of GDP-bound K-Ras^T50I^ (PDB: 7T1F). The WT K-Ras (K-Ras^WT^) and K-Ras^T50I^ structures are very similar, with a backbone root mean square deviation of 0.7 Å between them (Supplemental Figure 1D). Isolated on the solvent-exposed side of a β-sheet, the impact of the T50I substitution on the overall structure is minimal, and a direct structural link between T50 and E37 can be ruled out (Supplemental Figure 1, C and D). Despite substantial differences in the C-terminal hypervariable domains of H-Ras and K-Ras4b, the structures of K-Ras^T50I^-GDP and H-Ras^T50I^-GTP (10) are highly similar after accounting for the expected effects of GDP versus GTP binding.

We next asked if recombinant K-Ras^T50I^ has a reduced rate of intrinsic GTP hydrolysis or impaired GAP sensitivity, as commonly observed in oncogenic and Rasopathy K-Ras mutant proteins (1, 8, 9, 11, 32). Recombinant K-Ras^WT^, K-Ras^T50I^, oncogenic K-Ras^G12D^, and K-Ras^G12D,T50I^ proteins (residues 1–169) were generated and loaded with [^32^P] γ-GTP to assess intrinsic GTPase activity and sensitivity to the GAP domains of p120GAP and neurofibromin (32, 33). K-Ras^WT^ and K-Ras^T50I^ exhibited similar intrinsic GTP hydrolysis rates and sensitivity to GAP stimulation (Supplemental Figure 2, A and B). As expected, K-Ras^G12D^ showed impaired intrinsic GTP hydrolysis and resistance to GAPs, which were unaffected by a secondary T50I substitution (Supplemental Figure 2, A and B). Together, these data support the idea that T50I enhances MAPK signaling through a non-GTPase mechanism.

A broadly applicable model system for investigating how *KRAS* mutations modulate cellular phenotypes and MAPK signaling would ideally be characterized by (a) a tissue context or cell lineage in which Ras proteins regulate cell fate decisions and *RAS* mutations occur in cancer, (b) dependence on MAPK signaling for survival and proliferation, and (c) the flexibility to inducibly express individual mutant K-Ras proteins in the presence of endogenous levels of H-Ras and N-Ras. The fms like tyrosine kinase 3–mutant (*FLT3*-mutant) acute myeloid leukemia (AML) cell line MOLM-13 fulfills these criteria. Somatic *NRAS* and *KRAS* mutations are prevalent in AML, become undetectable during remission, and reappear or are replaced by a different signaling mutation at relapse (34–36). *NRAS/KRAS* mutations frequently emerge at relapse in patients treated with clinical FLT3 inhibitors, and Ras oncoprotein expression rescues *FLT3*-mutant leukemia cell lines from drug-induced apoptosis (37). We reasoned that MOLM-13 cells could be engineered as a “switchable” system by using the clinical inhibitor quizartinib (AC220) to suppress oncogenic FLT3 signaling and then assessing the biologic and biochemical consequences of expressing different Ras proteins at physiologic levels (38).

**Figure 1:**
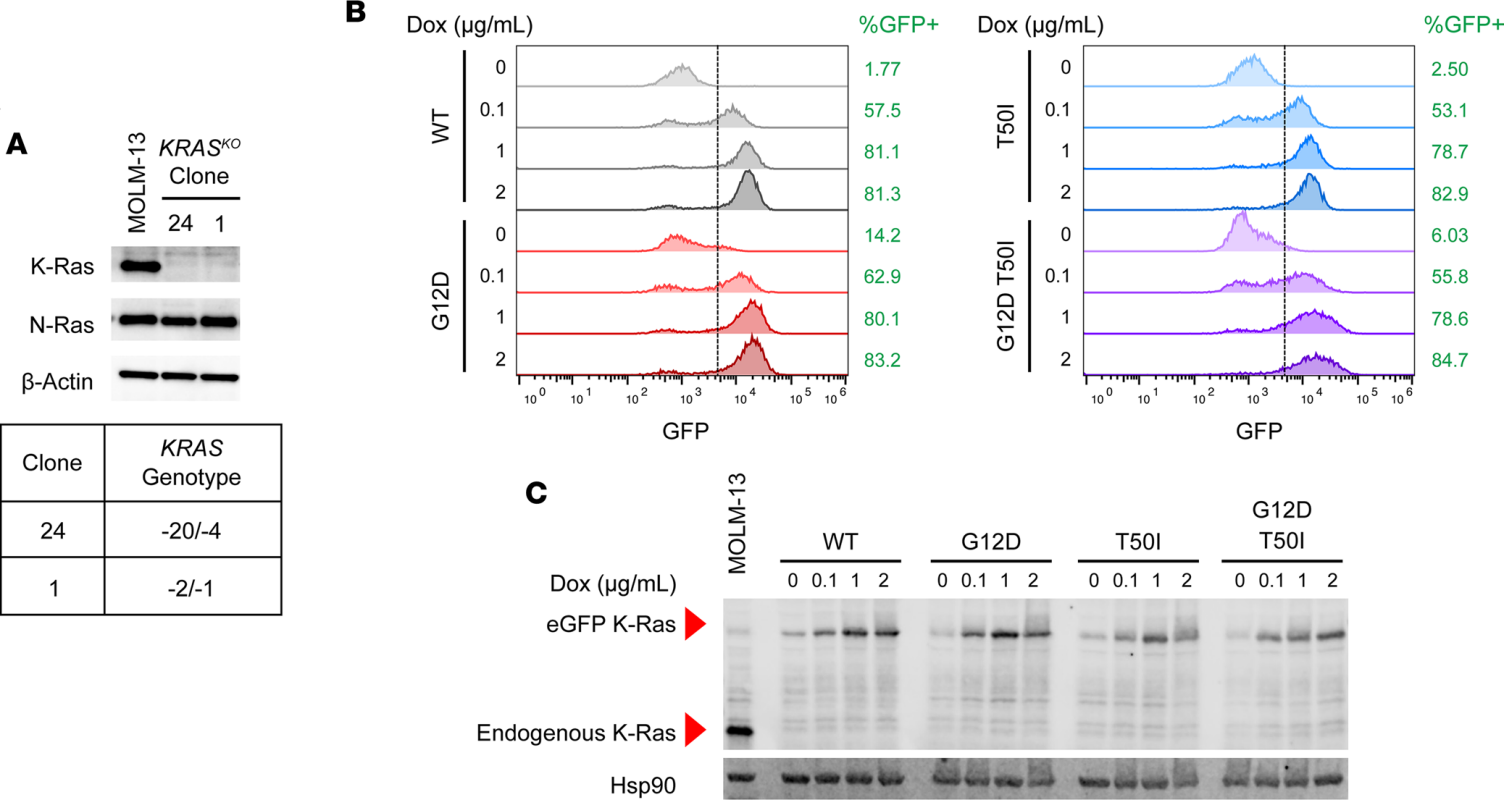
Regulatable K-Ras expression in FLT3-dependent MOLM-13 *KRAS^KO^* cells. (**A**) *KRAS^KO^* clones 1 and 24 were generated from MOLM-13 cells by CRISPR/Cas9-mediated editing. Western blotting verified loss of K-Ras protein expression (top), and Sanger sequencing confirmed biallelic frameshift insertion–deletion mutations in both clones (bottom). (**B**) Flow cytometry analysis of *KRAS^KO^* clone 24 cells expressing individual dox-inducible EGFP–K-Ras fusion proteins. Exposure to 2 μg/mL dox consistently induced EGFP–K-Ras expression in greater than 80% of events analyzed in clone 24 cells expressing EGFP–K-Ras^WT^, EGFP–K-Ras^G12D^, EGFP–K-Ras^T50I^, or EGFP–K-Ras^G12D,T50I^. (**C**) Western blotting of lysates prepared from the clone 24 cells shown in **B** demonstrates comparable levels of EGFP–K-Ras in *KRAS^KO^* cells (~50 kDa due to addition of the EGFP cassette) and endogenous K-Ras (21 kDa) in parental MOLM-13 cells.

Accordingly, we performed CRISPR/Cas9 gene editing to generate independent MOLM-13 single-cell clones characterized by biallelic frameshift *KRAS* mutations and loss of K-Ras protein expression (Figure 1A; see complete unedited blots in the supplemental material). *KRAS*-knockout (*KRAS^KO^*) clones 1 and 24 displayed similar sensitivities to AC220 as parental MOLM-13 cells (Supplemental Figure 3, A and B). These cells were transduced with lentivirus to stably express doxycycline-inducible (dox-inducible) K-Ras proteins fused to an N-terminal–enhanced GFP (EGFP) cassette. We used the EGFP marker to isolate *KRAS^KO^* cells with similar EGFP–K-Ras levels and titrated the dox concentration to mimic endogenous K-Ras expression (Figure 1, B and C). Treatment with 2 μg/mL dox induced physiologic levels of WT and mutant EGFP–K-Ras protein expression in a high percentage of MOLM-13 *KRAS^KO^* cells (Figure 1, B and C, and Supplemental Figure 3C). MOLM-13 cells are dependent on oncogenic FLT3 signaling for survival and proliferation (37, 39). We assessed the ability of EGFP–K-Ras^WT^ and of individual K-Ras mutant proteins to inhibit apoptosis and maintain MAPK pathway activation upon exposure to a dose of AC220 (10 nM) that suppressed ERK phosphorylation. In addition to investigating *KRAS^KO^* cells expressing EGFP–K-Ras^WT^, EGFP-K-Ras^T50I^, and EGFP-K-Ras^G12D^, we generated isogenic clones expressing an E162L substitution that in silico structural analysis predicted would cooperate with T50I to promote Ras:Ras clustering (Supplemental Figure 4).

**Figure 2:**
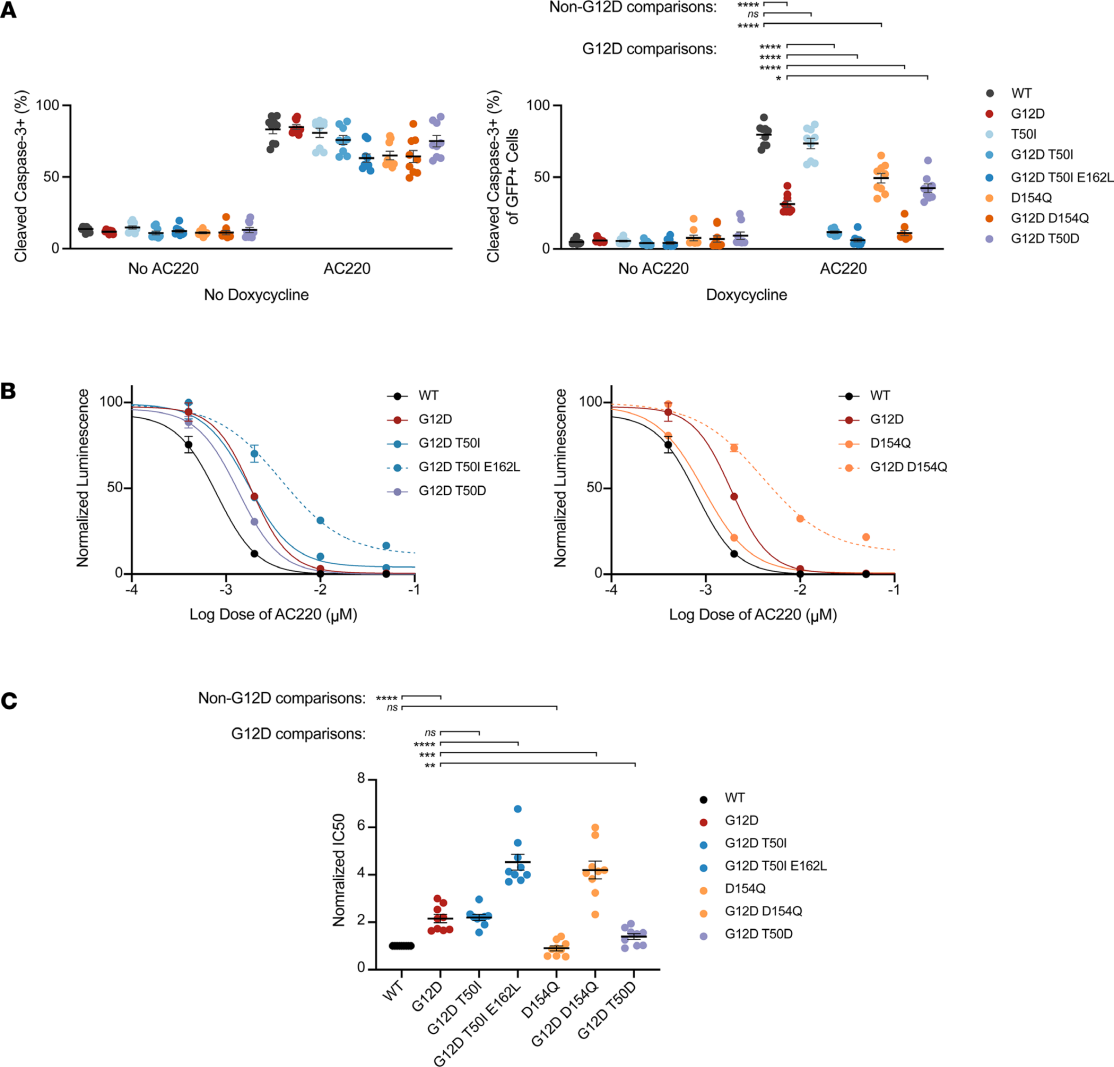
Mutant EGFP–K-Ras proteins have variable effects on CC3 induction that correlate with resistance to AC220 in MOLM-13 *KRAS^KO^* clone 24 cells. (**A**) Percentages of CC3^+^ clone 24 cells were measured by flow cytometry after exposure to DMSO (control) or 10 nM AC220 in the absence (left) or presence (right) of 2 μg/mL dox for 48 hours. AC220 efficiently induces apoptosis in the absence of dox-induced K-Ras expression, which is variably suppressed by expressing different K-Ras proteins. Aggregated CC3 results for clone 24 over 3 independent experiments each performed in technical triplicate. (**B**) CTG analysis of cells expressing the indicated K-Ras proteins that were exposed to 2 μg/mL dox and a range of AC220 concentrations for 72 hours. For clarity, data for T50 and D154 mutants are plotted separately. (**C**) Normalized IC_50_ values for 3 independent CTG experiments that were each performed in technical triplicate. Values shown are mean ± SEM. Multiple *t* tests were performed using the Holm-Šidák method to correct for multiple comparisons. Adjusted *P* values: ****P < 0.0001, ***P ≥ 0.0001 and < 0.001, **P ≥ 0.001 and < 0.01, *P ≥ 0.01 and < 0.05.

Expressing EGFP–K-Ras^G12D^ at endogenous levels enhanced the survival of MOLM-13 clone 24 cells upon AC220 treatment, as indicated by a reduction in the percentage of cells positive for cleaved caspase-3 (CC3) and EGFP (Figure 2A). By contrast, EGFP–K-Ras^WT^ and EGFP–K-Ras^T50I^ did not rescue these cells from AC220-induced death. This observation is consistent with the modest gain-of-function properties of T50I and other germline Rasopathy mutations (9–11, 32). However, introducing T50I as a second-site mutation significantly augmented the pro-survival activity of K-Ras^G12D^, and cells expressing a K-Ras^G12D,T50I,E162L^ triple-mutant protein exhibited the lowest percentage of CC3^+^ cells (Figure 2A). The observation that E162L augments the pro-survival activity of K-Ras^G12D,T50I^ is consistent with the idea that this mutation promotes Ras:Ras lateral assembly (Supplemental Figure 1A and Supplemental Figure 4). Similarly, studies in which we used CellTiter-Glo (CTG) to quantify metabolically active cells showed that K-Ras^G12D,T50I^ conferred partial resistance to AC220, which was further augmented in cells expressing K-Ras^G12D,T50I,E162L^ (Figure 2, B and C). CC3 and CTG analyses of MOLM-13 *KRAS^KO^* clone 1 corroborated the findings in clone 24 (Supplemental Figure 5, A and B).

Previous studies proposed a key role for D154 in alternative models of Ras dimerization and reported that a D-to-Q substitution impaired oncogenic activity and decreased MAPK pathway activation in mouse embryonic fibroblasts lacking endogenous *Hras*, *Nras*, and *Kras* expression (40, 41) (Supplemental Figure 6). This idea is difficult to reconcile with recently solved Ras:Raf-RBD-CRD co-crystal structures (17, 18) and studies of Raf-CRD binding to nanodiscs (21) showing that D154 is in close proximity to the PM when Ras is bound to Raf and would not accommodate another Ras molecule nearby. To address the biochemical and phenotypic consequences of expressing this mutation at physiologic levels in an isogenic system, we generated MOLM-13 *KRAS^KO^* cells expressing EGFP–K-Ras^D154Q^ or EGFP–K-Ras^G12D,D154Q^. Despite some interclonal variability, EGFP–K-Ras^D154Q^ expression had minimal overall effects on AC220-induced apoptosis (Figure 2, A–C, and Supplemental Figure 5, A and B). Surprisingly, D154Q enhanced the pro-survival activity of K-Ras^G12D^ as a second-site mutation (Figure 2A and Supplemental Figure 5A). Accordingly, MOLM-13 *KRAS^KO^* cells expressing EGFP–K-Ras^G12D,D154Q^ exhibited significantly higher AC220 IC_50_ values compared to cells expressing EGFP–K-Ras^G12D^ (Figure 2, B and C, and Supplemental Figure 5B). We conclude that D154Q has minimal effects on K-Ras function as a single mutation and unexpectedly augments the pro-survival activities of K-Ras^G12D^ in MOLM-13 *KRAS^KO^* cells.

To evaluate the possibility that any second-site substitution would nonspecifically alter K-Ras^G12D^ function, we generated *KRAS^KO^* cells expressing EGFP–K-Ras^G12D,T50D^. Structural modeling predicted that T50D would have limited effects on EGFP–K-Ras^G12D^ activity, which is what was observed (Figure 2, A–C, and Supplemental Figure 5). Furthermore, recombinant K-Ras^T50D^ proteins exhibited normal intrinsic GTP hydrolysis, confirming that this mutation does not alter GTPase activity (Supplemental Figure 2A).

**Figure 3:**
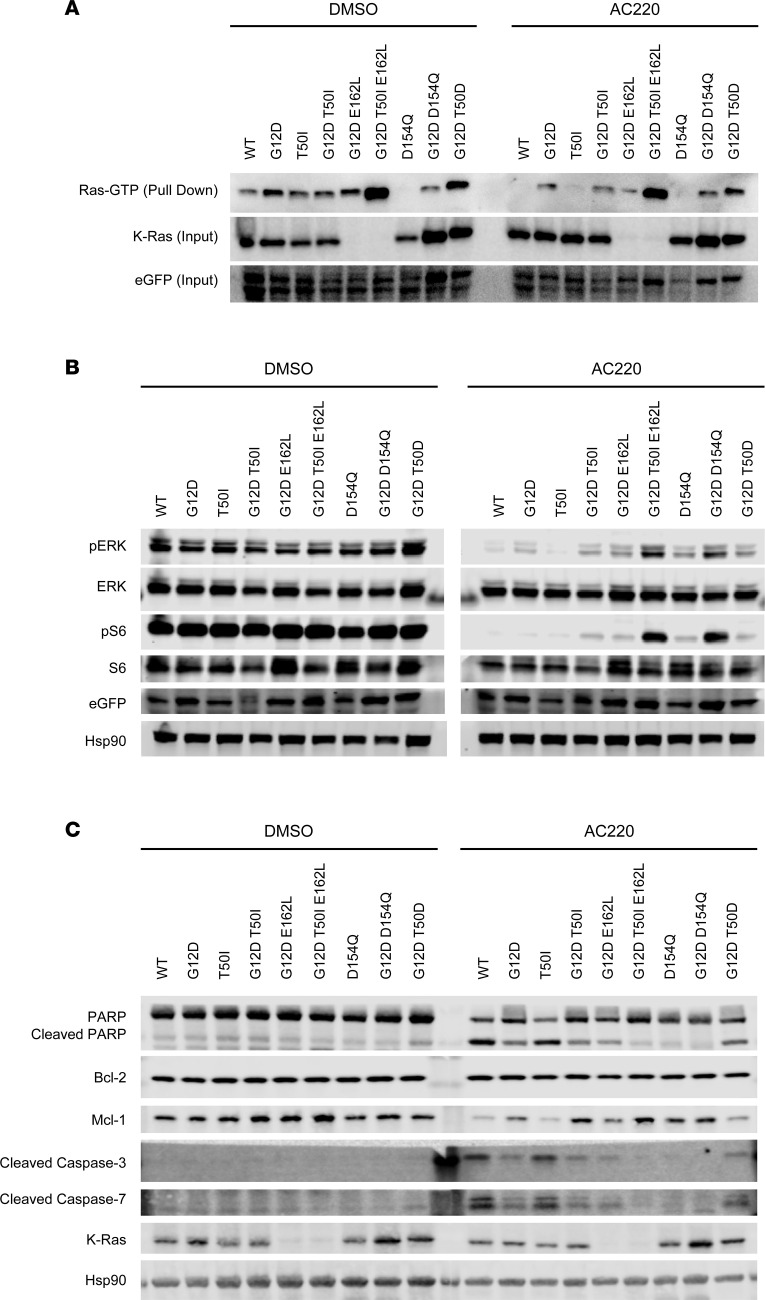
Biochemical analysis of MOLM-13 *KRAS^KO^* clone 24 cells showing variable effects of different EGFP–K-Ras proteins on Ras-GTP levels, ERK/S6 phosphorylation, and the expression of pro-survival and apoptotic proteins. (**A**) The RBD of Raf-1 was used to pull down Ras-GTP in clone 24 cells expressing the indicated EGFP–K-Ras proteins that were treated with dox for 24 hours and then exposed to either DMSO (left) or AC220 (10 nM; right) for 2 hours. (**B**) Western blotting to assess total and p-ERK and p-S6 levels in the same cells shown in **A**. (**C**) Western blotting showing PARP/cleaved PARP, Bcl-2, Mcl-1, and cleaved caspase-3/7 expression. Hsp90 is a loading control. The K-Ras Ab used in these experiments does not detect E162L mutant proteins, whereas the EGFP Ab detects all EGFP–K-Ras proteins. Each panel shows representative data from at least 2 independent experiments.

Basal Ras-GTP levels are elevated in MOLM-13 *KRAS^KO^* cells that express EGFP-K-Ras^G12D^ (Figure 3A, DMSO) and persist after exposure to AC220 (Figure 3A, AC220). By contrast, AC220 treatment efficiently suppressed Ras activation in cells expressing EGFP–K-Ras^WT^, EGFP–K-Ras^T50I^, or EGFP–K-Ras^D154Q^ (Figure 3A, right). Residues S235 and S236 of S6 ribosomal protein (S6) are phosphorylated downstream of both MAPK and PI3K/Akt in myeloid-lineage cells (42). Phosphorylated (p-) ERK1/2 and S6^S235/236^ levels were constitutively elevated in MOLM-13 cells due to oncogenic FLT3 signaling (Figure 3B, DMSO) and were fully suppressed by AC220 treatment in cells expressing EGFP–K-Ras^WT^, EGFP–K-Ras^T50I^, or EGFP–K-Ras^D154Q^ (Figure 3B, AC220). By contrast, cells expressing EGFP–K-Ras^G12D^ with and without additional mutations maintained variable degrees of ERK and S6 activation, which correlated with Ras-GTP levels (Figure 3, A and B). Similarly, the biochemical profiles of the full panel of MOLM-13 *KRAS^KO^* cells were concordant with functional data generated in CC3 and CTG assays (Figure 2, A–C; Figure 3, A and B; and Supplemental Figure 5).

Western blot analysis verified the changes in CC3 protein levels detected by flow cytometry in response to AC220 and dox treatment (Figure 3C). Additional markers of apoptosis including cleaved PARP, cleaved caspase-7, and annexin V were all robustly induced by AC220 treatment in clone 24 MOLM-13 *KRAS^KO^* cells. Consistent with the CC3 data, these responses were variably suppressed by dox-induced expression of K-Ras mutant proteins with K-Ras^WT^ and K-Ras^T50I^ similar to parental MOLM-13 cells, K-Ras^G12D^ and K-Ras^G12D,T50D^ characterized by modest effects, and K-Ras^G12D,D154Q^ and the K-Ras^G12D,T50I,E162L^ triple-mutant protein exhibiting potent anti-apoptotic activity (Figure 3C and Supplemental Figure 7). Whereas expression of the pro-survival protein Bcl-2 was unaffected by treatment with AC220 and/or dox, Mcl-1 was dynamically modulated, exhibiting markedly reduced expression in K-Ras^G12D,D154Q^ and K-Ras^G12D,T50I,E162L^ cells (Figure 3C). S-phase analysis using 5-ethynyl-2-deoxyuridine (EdU) labeling revealed profound cell cycle arrest upon AC220 exposure in parental MOLM-13 and MOLM-13 *KRAS^KO^* cells, which was partially reversed by dox-induced expression of different EGFP–K-Ras proteins in accordance with biochemical and IC_50_ data (Supplemental Figure 8). Together, these results verify the dependence of MOLM-13 cells on mutant *FLT3* expression for survival and proliferation (37, 38) and demonstrate the variable ability of different mutant K-Ras proteins to suppress these phenotypes.

An unresolved question in Ras biology is whether (and how) K-Ras^WT^ proteins antagonize the oncogenic activity of mutant K-Ras in cancer cells. An appealing explanation for this putative tumor suppressor activity is that K-Ras^WT^ proteins undergo rapid GTP hydrolysis, which destabilizes mutant K-Ras–GTP clusters and attenuates signal output (43–45). Recent studies demonstrated phenotypic tumor suppressor activity of K-Ras^WT^ in primary mouse AML cells and in human lung and colorectal cancer cell lines and also showed that loss of *KRAS^WT^* provided a fitness advantage and sensitized cells to MEK inhibition (40, 46). We explored this question by generating isogenic MOLM-13 *KRAS^KO^* clone 24 cells that stably express dox-inducible mCherry–K-Ras^WT^ and/or mCherry–K-Ras^G12D^ and then isolating EGFP^+^/mCherry^+^ cells by sorting to ensure uniform K-Ras expression upon dox treatment. mCherry–K-Ras^WT^ and mCherry–K-Ras^G12D^ had similar effects in *KRAS^KO^* cells as the corresponding EGFP-tagged proteins (Supplemental Figure 9, A and B). Interestingly, mCherry–K-Ras^G12D^ exhibited more potent pro-survival activity than did EGFP–K-Ras^G12D^ (Supplemental Figure 9, A and B), which correlated with higher p-ERK and p-S6 levels (Supplemental Figure 9C).

**Figure 4:**
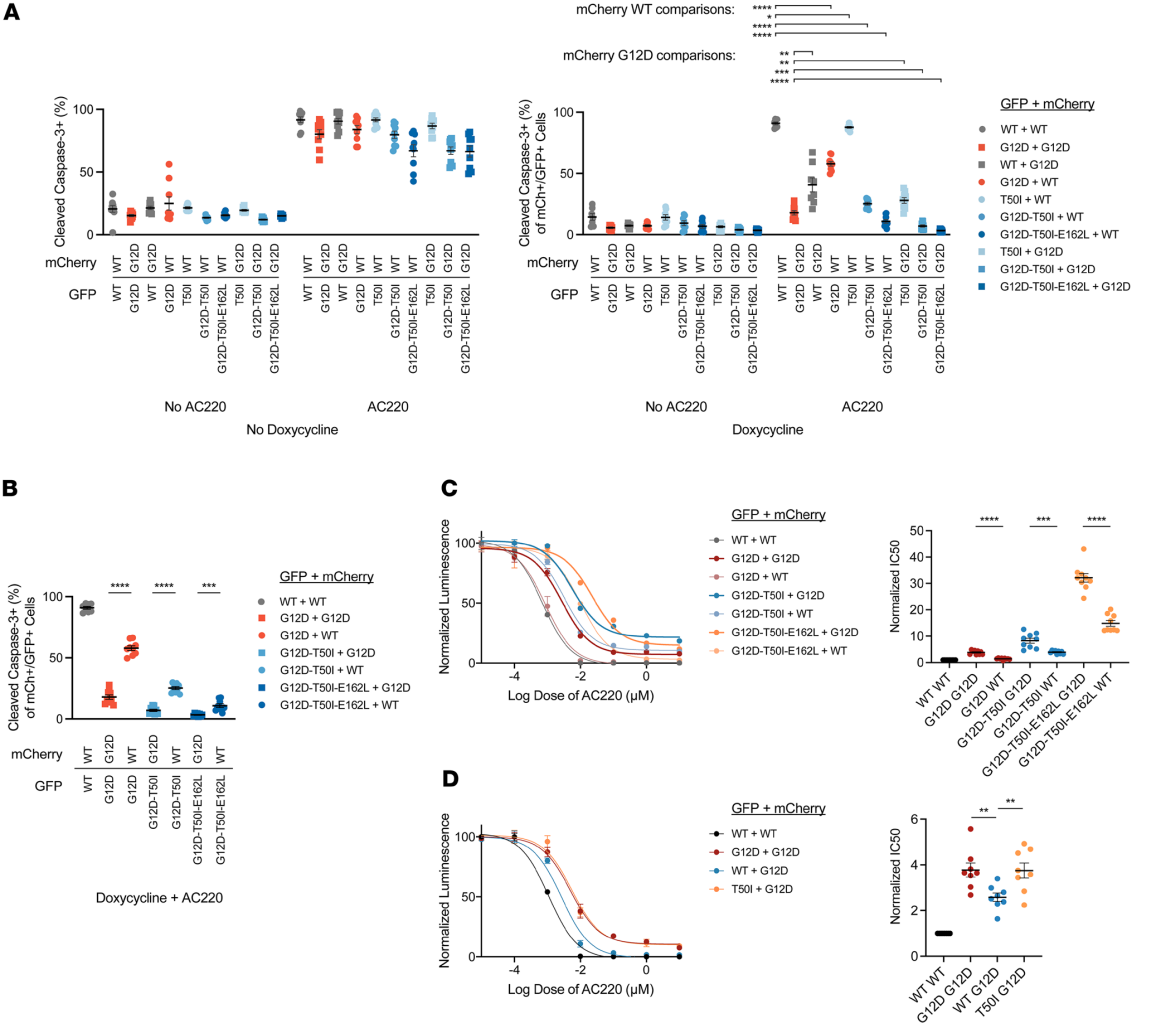
Coexpression of WT EGFP–K-Ras or mCherry–K-Ras in MOLM-13 *KRAS^KO^* clone 24 cells results in variable growth inhibition that correlates with the strength of individual mutant K-Ras proteins. (**A**) Expression of K-Ras^WT^ antagonizes the pro-survival activity of K-Ras^G12D^ with mCherry–K-Ras expression showing a more potent effect than EGFP–K-Ras. Clone 24 cells were treated with DMSO or 10 nM AC220 in the absence or presence of dox for 48 hours before flow cytometry–based analysis of CC3. Results are shown from 3 independent experiments each performed in technical triplicate. (**B**) Absolute increases in the percentage of CC3^+^ cells coexpressing EGFP–K-Ras proteins and either mCherry–K-RasWT or mCherry–K-RasG12D. (**C** and **D**) IC_50_ values were measured using CTG in cells coexpressing the indicated combinations of EGFP– and mCherry–K-Ras proteins. Normalized IC_50_ values for 3 independent CTG experiments that were each performed in technical triplicate. Values shown are mean ± SEM. Multiple *t* tests were performed using the Holm-Šidák method to correct for multiple comparisons. Adjusted *P* values: ****P < 0.0001, ***P ≥ 0.0001 and < 0.001, **P ≥ 0.001 and < 0.01, *P ≥ 0.01 and < 0.05.

Next, we coexpressed mCherry–K-Ras and EGFP–K-Ras in *KRAS^KO^* clone 24 cells and observed similar phenotypes as with either protein alone. Specifically, cells that coexpressed EGFP–K-Ras^WT^/mCherry–K-Ras^WT^ remained sensitive to AC220 treatment, whereas approximately 90% of cells coexpressing EGFP–K-Ras^G12D^/mCherry–K-Ras^G12D^ were resistant to apoptosis (Figure 4A). Furthermore, EGFP–K-Ras^WT^ and mCherry–K-Ras^WT^ proteins partially antagonized the anti-apoptotic activity of EGFP–K-Ras^G12D^ with mCherry–K-Ras^WT^ showing more potent effects than did EGFP–K-Ras^WT^ (Figure 4A). Coexpressing mCherry–K-Ras^WT^ with either EGFP–K-Ras^G12D,T50I^ or EGFP–K-Ras^G12D,T50I,E162L^ only modestly reduced the potent pro-survival activities of these proteins (Figure 4B). Because of the low levels of apoptosis in cells expressing EGFP–K-Ras^G12D,T50I^ and EGFP–K-Ras^G12D,T50I,E162L^, we performed CTG analyses to compare the effects of coexpressing either mCherry–K-Ras^WT^ or mCherry–K-Ras^G12D^. Consistent with the CC3 data, AC220 IC_50_ values were uniformly lower in cells expressing mCherry–K-Ras^WT^ versus mCherry–K-Ras^G12D^ (Figure 4C). Interestingly, coexpressing mCherry–K-Ras^G12D^ with EGFP–K-Ras^T50I^ or EGFP–K-Ras^G12D^ increased calculated AC220 IC_50_ values to a similar extent, which supports an unexpected trans effect of the T50I mutation in promoting resistance (Figure 4D). The biochemical effects of coexpressing various EGFP– and mCherry–K-Ras proteins correlated with the results of CC3 and CTG assays (Supplemental Figure 10).

Together, these data indicate that isogenic MOLM-13 *KRAS^KO^* cells are a robust system for interrogating the putative tumor suppressor activity of K-Ras^WT^ proteins. Notably, mutant K-Ras proteins that potently antagonized apoptosis and maintained effector activation upon AC220 exposure were less sensitive to inhibition by K-Ras^WT^ coexpression (Figure 4, A–C).

## Discussion

Although key structural details of the assembly of Ras:Raf signaling complexes in solution and at the PM have emerged recently (13, 17, 18), much remains to be learned regarding Ras lateral assembly and how this might affect Raf activation. Crystal structures of H-Ras^T50I^ and K-Ras4b^T50I^ and the normal intrinsic and GAP-stimulated GTP hydrolysis of recombinant K-Ras4b^T50I^ strongly support a non-GTPase mechanism for increased signal output, as suggested by others (10). Accordingly, we reasoned that interrogating T50I and additional amino acid substitutions implicated in the putative Ras:Ras interface would provide functional insights relevant to recent models of Raf activation at the PM (15, 17, 18, 40, 41, 47). The general approach described here is applicable for investigating Ras functions in other cancer cell line models characterized by driver kinase mutations such as *EGFR-*mutant lung adenocarcinoma and *BRAF*-mutant melanoma cell lines.

Our data showing that K-Ras^G12D^ protects MOLM-13 *KRAS^KO^* cells from apoptosis upon AC220 exposure align with findings from studies of human patients with AML, showing that *NRAS* and *KRAS* mutations cause adaptive resistance to clinical FLT3 inhibitors (37). Whereas K-Ras^T50I^ had minimal effects as a single mutation, this substitution potently augmented the activity of K-Ras^G12D^ in phenotypic assays. This observation is consistent with extensive data indicating that germline *NRAS* and *KRAS* mutations are less activated than somatic oncogenic mutations (9–11, 32). T50I enhances the oncogenic activity of K-Ras^G12D^ by increasing MAPK signaling, explaining the ability of this mutation to rescue impaired Raf binding by K-Ras^G12D,E37G^ (24). Lateral clustering of K-Ras is hypothesized to enhance Raf dimerization and MAPK pathway activation (43–45). On the basis of structural modeling, we predicted that a third-site E162L mutation might confer additional stability to K-Ras^G12D,T50I^ by increasing the “stickiness” of the Ras:Ras interaction and thereby augmenting clustering. Our studies of isogenic MOLM-13 *KRAS^KO^* cells expressing K-Ras^G12D,T50I,E162L^ support this hypothesis and align with the asymmetric Ras:Ras assembly model presented in Supplemental Figure 1.

**Figure 5:**
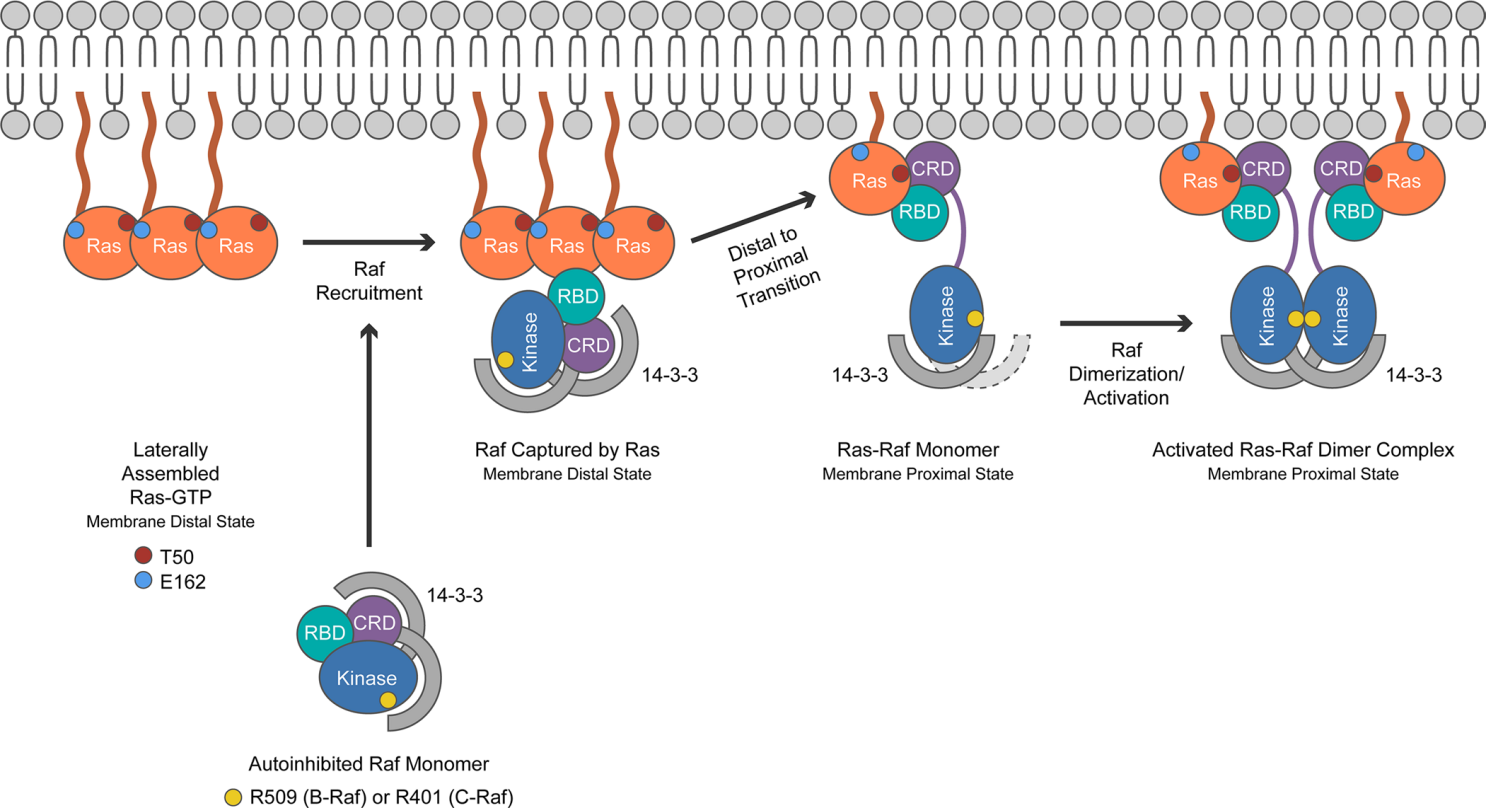
Potential impact of T50I in the context of recent models of Raf recruitment to the plasma membrane and activation by Ras-GTP. In accordance with the data of Van et al. (15, 17, 18, 47), T50I and E162L mutations could enhance the lateral assembly of Ras-GTP in the membrane-distal state, thereby increasing interaction avidity and range as “bait” to engage the RBD of Raf. Upon binding to Raf, the interswitch region of Ras, which includes T50, dissociates from neighboring Ras molecules and binds the Raf-CRD. Ras binding to the Raf-CRD has been shown to disassemble the auto-inhibited state of Raf, with the exposed basic α4/α5 side chains of Ras acting together with the Raf-CRD as lipid “adhesives” that facilitate transition of the Ras:Raf monomer to a plasma membrane–proximal state. When viewed in the context of recently solved co-crystal structures, a T50I mutation could increase signal output by stabilizing the interaction of Ras with the Raf-CRD at the PM. Kinase domain–exposed Raf is then poised to attract and integrate a second Ras:Raf monomer to form a productive signaling complex.

Informed by published data and models of Ras activation at the PM proposed by other investigators (15, 17, 18, 47), we speculate that T50I and E162L enhance stepwise Raf recruitment and activation by promoting G-domain–mediated Ras:Ras lateral clustering and increasing local concentrations of K-Ras with a spatial organization that favors Raf binding and activation with subsequent formation (“casting”) of a membrane-proximal signaling complex likely facilitated by Ras binding to the Raf-CRD as well as by the association of the Raf-CRD with the PM. Accordingly, we hypothesize that a T50I or E162L mutation increases hydrophobicity at the Ras surface, thereby promoting lateral assembly through an asymmetric interaction with another K-Ras molecule and subsequent Raf recruitment and activation (Figure 5).

Alternatively, and consistent with recently solved co-crystal structures of Ras bound to the Raf-RBD-CRD (17, 18), isoleucine 50 may stabilize the interaction between Ras and the Raf-CRD at the core of the Ras:Raf signaling complex upon formation of a stable Ras:Raf membrane-proximal monomer (Figure 5). Specifically, substitution of T50 with the bulkier isoleucine would allow direct hydrophobic interactions with both F141 and F163 of Raf that could enhance binding (Supplemental Figure 11). Because the affinity between Ras and the Raf-CRD is low, even a moderate improvement in binding might potentiate MAPK signaling. This idea is consistent with predictions that efficient Raf activation by Ras requires both a high-affinity interaction with the RBD and low-affinity binding to the CRD (19, 20). The possibilities that T50I augments asymmetric Ras lateral clustering and enhances binding to the Raf-CRD are not mutually exclusive, because the former occurs before Raf recruitment and the latter is dependent on formation of a membrane-proximal Ras:Raf monomer followed by Raf dimerization. Direct interactions between individual K-Ras molecules are not essential in this model, consistent with recent studies of Ras dimerization (48) (Figure 5).

In summary, we developed a versatile and disease-relevant system for interrogating the consequences of expressing mutant K-Ras proteins at physiologic levels. Using a T50I mutation found in Rasopathy developmental disorders and that overcomes the deleterious effects of an E37G substitution in leukemogenesis as a starting point for investigating non-GTPase mechanisms of Ras/MAPK activation, we integrated functional and biochemical data with recently solved, multicomponent, co-crystal structures. Together, these studies support the idea that productive MAPK activation by K-Ras is regulated by higher order complex formation and lateral assembly at the PM. The system described here will facilitate studies addressing key biologic questions such as isoform-selective Raf activation by Ras (47) and the tumor suppressor activity of WT Ras while also unveiling new therapeutic opportunities for targeting aberrant Ras/Raf/MAPK signaling in Rasopathy disorders and cancer. Furthermore, this approach can be extended to generate isogenic models for evaluating the specificity and inhibitory activity of next-generation chemical inhibitors of common oncogenic Ras proteins and for discovering how single- and second-site mutations alter sensitivity to these drugs (49, 50).

## Methods

### MOLM-13 cell line.

Early-passage MOLM-13 cells (DSMZ) were cultured in RPMI media (HyClone) containing 10% FBS (Corning), 1% penicillin-streptomycin (Thermo Fisher), and 1% GlutaMAX (Thermo Fisher).

### Ribonucleoprotein-based editing.

We incubated at 37°C for 30 minutes 160 μM CRISPR RNA and 160 μM trans-activating CRISPR RNA (Dharmacon) that were resuspended in 10 mM Tris pH 7.4 buffer (GE Healthcare). An equal volume of Cas9 (MacroLab Core Facility, UC Berkeley) was added and incubated at 37°C for 15 minutes. The mixture with 5 μL of 100 μM enhancer template (IDT) was added to 2 × 10^6^ MOLM-13 cells for nucleofection. Nucleofection was performed with SF Cell Line 4D-Nucleofector X Kit S and code DJ-110 (4D-Nucleofector X Unit; Lonza).

### Single-cell clone generation.

We plated 1 cell/well 5 days after nucleofection to isolate single-cell clones, which were confirmed for *KRAS* KO by genomic DNA isolation, PCR amplification and purification, and Sanger sequencing analysis (GENEWIZ; Synthego ICE).

### Plasmids, cloning, and mutagenesis.

The plasmids pCW57.1 (Addgene plasmid 41393), pDONR223 *KRAS* *WT* (Addgene plasmid 81751), and pDONR223 *KRAS* *G12D* (Addgene plasmid 81651) were used to generate dox-inducible *KRAS* constructs with Gateway LR Clonase enzyme mix (Thermo Fisher). EGFP was Gibson cloned from FgH1tUTG (a gift from Catherine Smith at UCSF) to the N-terminus of *KRAS* on pCW57.1 *KRAS*. mCherry-*KRAS* constructs were generated using pCW57.1 EGFP*-KRAS* as backbone, and the mCherry sequence of pHR-SFFV-*KRAS*-dCas9-P2A-mCherry (a gift from Luke Gilbert at UCSF) was used to replace EGFP by Gibson cloning. *KRAS* mutagenesis on the aforementioned inducible vectors was performed with the QuikChange II XL Site-Directed Mutagenesis Kit (Agilent).

### Lentiviral transduction and cell sorting.

Lentiviral backbone, packaging vector (dR8.91), and envelope (pMD2G) were transfected into 293T lenti-X cells (Takara Bio) with TransIT-LT1 (Mirus Bio). Supernatant was collected 48 hours after transfection and applied to *KRAS^KO^* clones with polybrene for transduction. Cells were spin-infected at 800*g* for 2 hours at 37°C. After puromycin selection, cells were treated with 2 μg/mL dox (Sigma) for 24 hours and sorted for EGFP^+^, mCherry^+^, or double-positive cells. For clone 24, EGFP^+^ sorted cells were subsequently sorted to isolate a population that did not express EGFP in the absence of dox.

### Immunoblotting.

Whole-cell lysates were collected and 30 μg of protein per sample was used for immunoblotting. Hsp90 (BD 610418, clone 68) or β-actin (Cell Signaling Technology [CST] 4967) Abs were used for loading controls. Additional primary Abs used for detecting were as follows: KRAS (Sigma; WH0003845M1, clone 3B10-2F2; does not detect proteins with E162L mutation), EGFP (Thermo Fisher Scientific; 11575712, clone F56-6A1.2.3), mCherry (CST 43590, clone E5D8F), p-ERK1/2 (CST 4370, clone D13.14.4E), ERK1/2 (CST 9107, clone 3A7), p-S6 (CST 4858, clone D57.2.2E), S6 (CST 2317, clone 54D2), NRAS (Proteintech; 10724-1-AP, clone Ag1081), Bcl-2 (CST 3498, clone D17C4), Mcl-1 (CST 94296, clone D2W9E), PARP (CST 9532, clone 46D11), cleaved caspase-3 (CST 9661; cleavage adjacent to Asp175), and cleaved caspase-7 (CST 9491; cleavage adjacent to Asp198). Ras-GTP assays were performed with the Active RAS Pull-Down Kit (Thermo Fisher).

### CC3, annexin V, and EdU assays.

CC3 expression levels were assessed by flow cytometry after plating 20,000 cells/well in 96-well, round-bottom plates (Corning) and then treating them with or without 2 μg/mL dox (Sigma) and DMSO (Sigma) or 10 nM AC220 (Selleckchem) for 48 hours. Fixed and permeabilized cells were stained with a CC3 Ab (BD Horizon; catalog 560627) for 1 hour and analyzed on the Attune NxT flow cytometer (Thermo Fisher) at the UCSF HDFCCC LCA Core Facility. Annexin V and EdU were similarly assessed by flow cytometry after treating with dox and AC220 for 48 and 24 hours, respectively, then following the staining protocols as specified by their respective manufacturers: Annexin V Apoptosis Detection Kit (BD 556547) and Click-iT Plus EdU Flow Cytometry Assay Kit (Thermo Fisher C10634).

### Cell viability analysis.

We plated 1,000 cells/well in opaque, 96-well, flat-bottom plates (PerkinElmer), then treated them with or without 2 μg/mL dox (Sigma) and AC220 (Selleckchem) for 72 hours. Cell viability was analyzed with CTG (Promega).

### Recombinant K-Ras GTP-hydrolysis assay.

Constructs to make recombinant K-Ras proteins (residues 1–169) were gene synthesized and subcloned into pET-29b^+^ bacterial expression vectors with an N-terminal His6 fusion tag followed by a tobacco etch virus protease cleavage site (ENLYFQG) by GenScript. Resulting plasmids were transformed into BL21 CodonPlus (DE3) RIPL *E*. *coli* strain to generate recombinant proteins. Intrinsic and GAP-stimulated GTP hydrolysis assays have been described previously (51, 52).

### Crystallization and structure determination.

K-Ras^T50I^ protein (38.5 mg/mL) was set up using the sitting drop method at 4°C. Protein was diluted 1:1 with mother liquor (0.2 M calcium chloride, 20% PEG3,350). Crystal was soaked in a solution containing the mother liquor with 20% (vol/vol) glycerol, followed by flash-freezing. X-ray diffraction data were collected at beamline 8.3.1 at the Advanced Light Source (Lawrence Berkeley National Laboratory). Data were processed and scaled using MOSFLM and SCALA in the CCP4 package (Supplemental Table 1). Structure was solved by molecular replacement using the structure of a GDP-bound K-Ras^G12C^ protein (PDB: 4LDJ) as a starting mode and refined until there was no further improvement. Structural biology data have been deposited in the Worldwide PDB (PDB: 7T1F).

### Statistics.

Statistical analyses were performed using GraphPad Prism 8. Multiple *t* tests were performed using the Holm-Šidák method to correct for multiple comparisons. In the figures, the degree of significance is denoted by the number of asterisks (****P < 0.0001, ***P ≥ 0.0001 and < 0.001, **P ≥ 0.001 and < 0.01, *P ≥ 0.01 and < 0.05, NS ≥ 0.05). All data indicate the mean ± SEM.

### Data availability.

Values for all data points found in graphs are in the Supporting Data Values file.

## Author contributions

PYC, GB, CZ, KS, and YZ conceptualized the study and designed the experimental methodology; PYC, BJH, MH, CB, WW, GB, and KS performed the cellular and biochemical investigations; HC, DM, and AK generated recombinant proteins; BM performed crystallography experiments; CZ and YZ performed structural analyses; BJH performed statistical analyses; PYC, BJH, GB, CZ, KS, and YZ wrote the paper; GB, CZ, KS, and YZ supervised the project; and PYC, BJH, and KS handled the project administration and funding. PYC initiated this project while working as a postdoctoral scholar in the laboratory of KS, and generated key reagents and much of the data before taking a scientist position at Plexxikon. At that time, BJH assumed overall responsibility for and completed the project while working in the laboratory of KS.

## Supplementary Material

Supplemental data

Supporting data values

## Version 1 09/08/2023

Electronic publication

## References

[1] Simanshu DK (2017). RAS proteins and their regulators in human disease. Cell.

[2] Canon J (2019). The clinical KRAS(G12C) inhibitor AMG 510 drives anti-tumour immunity. Nature.

[3] Hallin J (2020). The KRAS^G12C^ inhibitor MRTX849 provides insight toward therapeutic susceptibility of KRAS-mutant cancers in mouse models and patients. Cancer Discov.

[4] Zhao Y (2021). Diverse alterations associated with resistance to KRAS(G12C) inhibition. Nature.

[5] Awad MM (2021). Acquired resistance to KRAS^G12C^ inhibition in cancer. N Engl J Med.

[6] Wang T (2017). Gene essentiality profiling reveals gene networks and synthetic lethal interactions with oncogenic ras. Cell.

[7] Lou K (2019). KRAS^G12C^ inhibition produces a driver-limited state revealing collateral dependencies. Sci Signal.

[8] Schubbert S (2007). Hyperactive Ras in developmental disorders and cancer. Nat Rev Cancer.

[9] Schubbert S (2007). Biochemical and functional characterization of germ line KRAS mutations. Mol Cell Biol.

[10] Cirstea IC (2010). A restricted spectrum of NRAS mutations causes Noonan syndrome. Nat Genet.

[11] Gremer L (2011). Germline KRAS mutations cause aberrant biochemical and physical properties leading to developmental disorders. Hum Mutat.

[12] Sarkozy A (2009). Germline BRAF mutations in Noonan, LEOPARD, and cardiofaciocutaneous syndromes: molecular diversity and associated phenotypic spectrum. Hum Mutat.

[13] Park E (2019). Architecture of autoinhibited and active BRAF-MEK1-14-3-3 complexes. Nature.

[14] Spencer-Smith R (2022). RASopathy mutations provide functional insight into the BRAF cysteine-rich domain and reveal the importance of autoinhibition in BRAF regulation. Mol Cell.

[15] Van QN (2020). Uncovering a membrane-distal conformation of KRAS available to recruit RAF to the plasma membrane. Proc Natl Acad Sci U S A.

[16] Kondo Y (2019). Cryo-EM structure of a dimeric B-Raf:14-3-3 complex reveals asymmetry in the active sites of B-Raf kinases. Science.

[17] Tran TH (2021). KRAS interaction with RAF1 RAS-binding domain and cysteine-rich domain provides insights into RAS-mediated RAF activation. Nat Commun.

[18] Cookis T, Mattos C (2021). Crystal structure reveals the full ras-raf interface and advances mechanistic understanding of Raf activation. Biomolecules.

[19] Brtva TR (1995). Two distinct Raf domains mediate interaction with Ras. J Biol Chem.

[20] Drugan JK (1996). Ras interaction with two distinct binding domains in Raf-1 may be required for Ras transformation. J Biol Chem.

[21] Fang Z (2020). Multivalent assembly of KRAS with the RAS-binding and cysteine-rich domains of CRAF on the membrane. Proc Natl Acad Sci U S A.

[22] Sakai E (2016). TP53 mutation at early stage of colorectal cancer progression from two types of laterally spreading tumors. Cancer Sci.

[23] Siroy AE (2015). Beyond BRAF(V600): clinical mutation panel testing by next-generation sequencing in advanced melanoma. J Invest Dermatol.

[24] Shieh A (2013). Defective K-Ras oncoproteins overcome impaired effector activation to initiate leukemia in vivo. Blood.

[25] Chen M (2016). Ras dimer formation as a new signaling mechanism and potential cancer therapeutic target. Mini Rev Med Chem.

[26] Lin WC (2014). H-Ras forms dimers on membrane surfaces via a protein-protein interface. Proc Natl Acad Sci U S A.

[27] Muratcioglu S (2015). GTP-dependent K-Ras dimerization. Structure.

[28] Prakash P (2017). Computational and biochemical characterization of two partially overlapping interfaces and multiple weak-affinity K-Ras dimers. Sci Rep.

[29] Abankwa D (2008). A novel switch region regulates H-ras membrane orientation and signal output. EMBO J.

[30] Fetics SK (2015). Allosteric effects of the oncogenic RasQ61L mutant on Raf-RBD. Structure.

[31] Rodriguez-Viciana P (1997). Role of phosphoinositide 3-OH kinase in cell transformation and control of the actin cytoskeleton by Ras. Cell.

[32] Schubbert S (2006). Germline KRAS mutations cause Noonan syndrome. Nat Genet.

[33] Bollag G, McCormick F (1995). Intrinsic and GTPase-activating protein-stimulated Ras GTPase assays. Methods Enzymol.

[34] Bolouri H (2018). The molecular landscape of pediatric acute myeloid leukemia reveals recurrent structural alterations and age-specific mutational interactions. Nat Med.

[35] Lindsley RC, Ebert BL (2013). The biology and clinical impact of genetic lesions in myeloid malignancies. Blood.

[36] Papaemmanuil E (2016). Genomic classification and prognosis in acute myeloid leukemia. N Engl J Med.

[37] McMahon CM (2019). Clonal selection with RAS pathway activation mediates secondary clinical resistance to selective FLT3 inhibition in acute myeloid leukemia. Cancer Discov.

[38] Zarrinkar PP (2009). AC220 is a uniquely potent and selective inhibitor of FLT3 for the treatment of acute myeloid leukemia (AML). Blood.

[39] Grundy M (2010). The FLT3 internal tandem duplication mutation is a secondary target of the aurora B kinase inhibitor AZD1152-HQPA in acute myelogenous leukemia cells. Mol Cancer Ther.

[40] Ambrogio C (2018). KRAS dimerization impacts MEK inhibitor sensitivity and oncogenic activity of mutant KRAS. Cell.

[41] Mysore VP (2021). A structural model of a Ras-Raf signalosome. Nat Struct Mol Biol.

[42] Van Meter ME (2007). K-RasG12D expression induces hyperproliferation and aberrant signaling in primary hematopoietic stem/progenitor cells. Blood.

[43] Cho KJ, Hancock JF (2013). Ras nanoclusters: a new drug target?. Small GTPases.

[44] Lin YJ, Haigis KM (2018). Brother’s keeper: wild-type mutant K-Ras dimers limit oncogenesis. Cell.

[45] Nan X (2013). Single-molecule superresolution imaging allows quantitative analysis of RAF multimer formation and signaling. Proc Natl Acad Sci U S A.

[46] Burgess MR (2017). KRAS allelic imbalance enhances fitness and modulates map kinase dependence in cancer. Cell.

[47] Terrell EM (2019). Distinct binding preferences between Ras and Raf family members and the impact on oncogenic Ras signaling. Mol Cell.

[48] Simanshu DK (2023). Consensus on the RAS dimerization hypothesis: Strong evidence for lipid-mediated clustering but not for G-domain-mediated interactions. Mol Cell.

[49] Feng S (2022). A saturation mutagenesis screen uncovers resistant and sensitizing secondary *KRAS* mutations to clinical KRAS^G12C^ inhibitors. Proc Natl Acad Sci U S A.

[50] Hallin J (2022). Anti-tumor efficacy of a potent and selective non-covalent KRAS^G12D^ inhibitor. Nat Med.

[51] Bollag G, McCormick F (1991). Differential regulation of rasGAP and neurofibromatosis gene product activities. Nature.

[52] Bollag G (1996). Loss of *NF*1 results in activation of the Ras signaling pathway and leads to aberrant growth in murine and human hematopoietic cells. Nat Genet.

