# Supplementary material for: Structural and functional analyses of a germline KRAS T50I mutation provide insights into Raf activation
Source: JCI Insight. 2023 Sep 8;8(17):e168445. doi: 10.1172/jci.insight.168445 (PMC10544224; doi:10.1172/jci.insight.168445)
Supplement: Supplemental data [file jciinsight-8-168445-s265.pdf]

Supplemental Figures and Tables

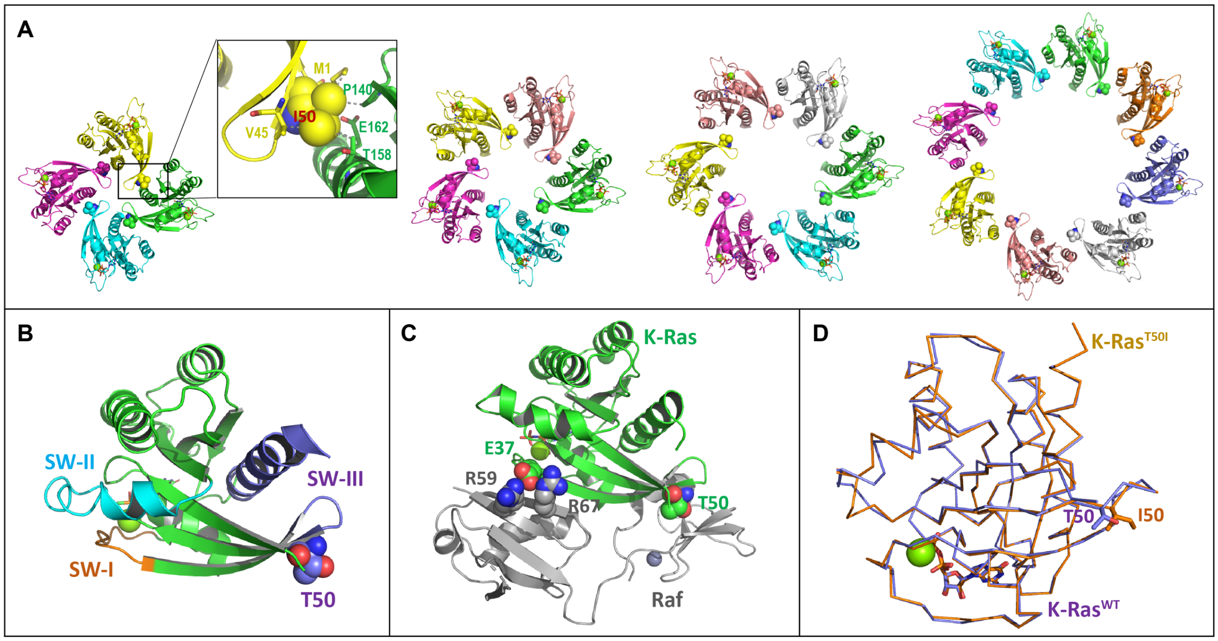

Supplemental Figure 1. Crystal structure of K-RasT50I and potential effects of T50I substitution on Ras:Raf and Ras:Ras binding. (A) K-Ras tetramers (PDB: 6Q21) and putative higher-order oligomers constructed using the same asymmetric protein-protein interface mediated by the inter-switch β2-β3 region. T50I is predicted to stabilize the interface by adding hydrophobic interactions with the other interface residues (M1 and P140). E162L is expected to further enhance hydrophobic packing. (B) Cartoon representation of K-Ras highlighting Switch I, II, and III regions. (C) Published structure of the K-Ras:Raf1-RBD-CRD complex (PDB:6XI7) with K-Ras residues E37 and T50, as well as Raf1 residues R59 and R67 highlighted in sphere mode. E37 forms cross β-strand interactions with Raf1 R59 and R67, whereas T50 faces the Raf1-CRD. (D) Overlay of WT and T50I K-Ras-GDP structures shows minimal global structural perturbation by the T50I substitution.

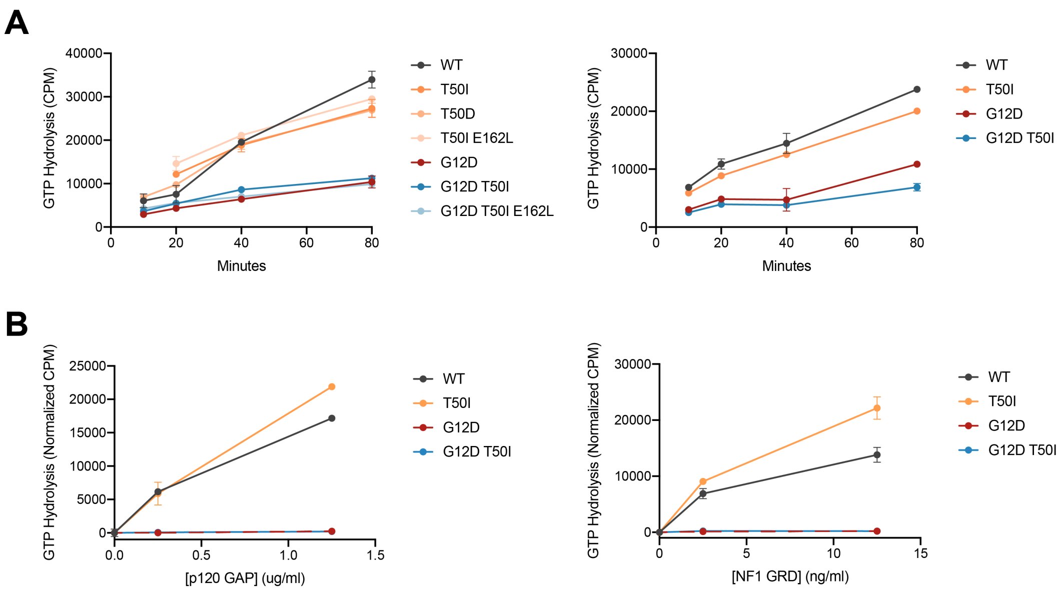

Supplemental Figure 2. A T50I substitution does not alter GTP hydrolysis of recombinant K-Ras proteins. (A) Intrinsic GTP hydrolysis of wild type (WT) and mutant K-Ras proteins showing replicate data (mean +/- SEM) generated in two independent experiments. The data plots represent liquid scintillation counts per minute (CPM) of phosphate molecules released from K-Ras proteins loaded with γ-P^{32} GTP. (B) Sensitivity of WT and mutant K-Ras proteins to GAP-related domain (GRD) peptides of p120 GAP or neurofibromin (NF1). The data plots indicate liquid scintillation CPM of phosphate molecules released by K-Ras proteins that were loaded with γ-P^{32} GTP and incubated for 8 minutes. These assays were performed at room temperature over a range of p120 GAP GRD or NF1 GRD concentrations and depict replicate data (mean ± SEM) obtained from two independent experiments.

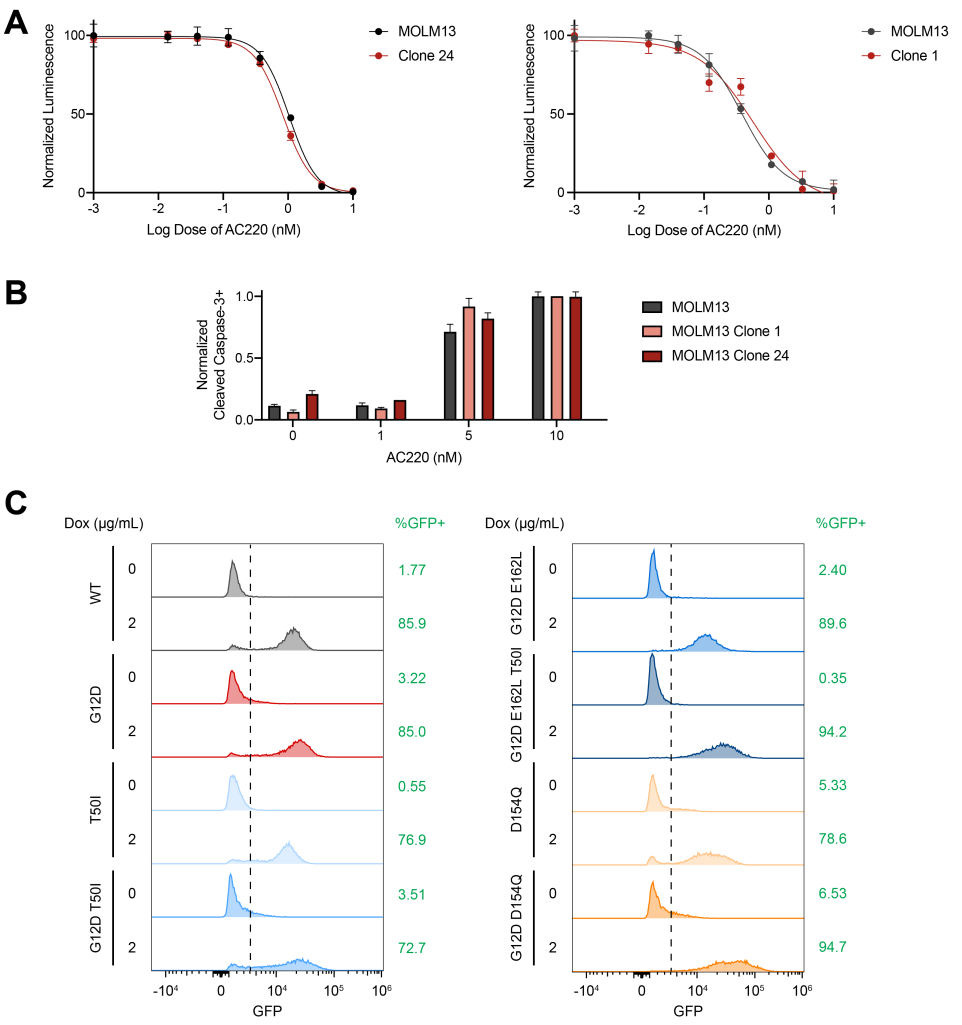

Supplemental Figure 3. Regulated expression of K-Ras proteins in MOLM-13 *KRAS*^{KO} clones 1 and 24. (A) Independent *KRAS* knockout clones 1 and 24 exhibit similar sensitivity to AC220 compared to parental MOLM-13 cells based on CellTiter-Glo assays. (B) Like parental MOLM-13 cells, independent *KRAS* knockout clones undergo apoptosis when exposed to AC220 (1 to 10 nM) as assessed by cleaved caspase 3 (CC3) staining. Shown are mean ± SEM for triplicate samples. (C) Flow cytometry analysis of clone 24 cells expressing individual dox-inducible eGFP-tagged K-Ras fusion proteins. Treatment with 2 µg/mL of dox consistently induced robust levels of wild-type (WT) and mutant eGFP-K-Ras protein expression.

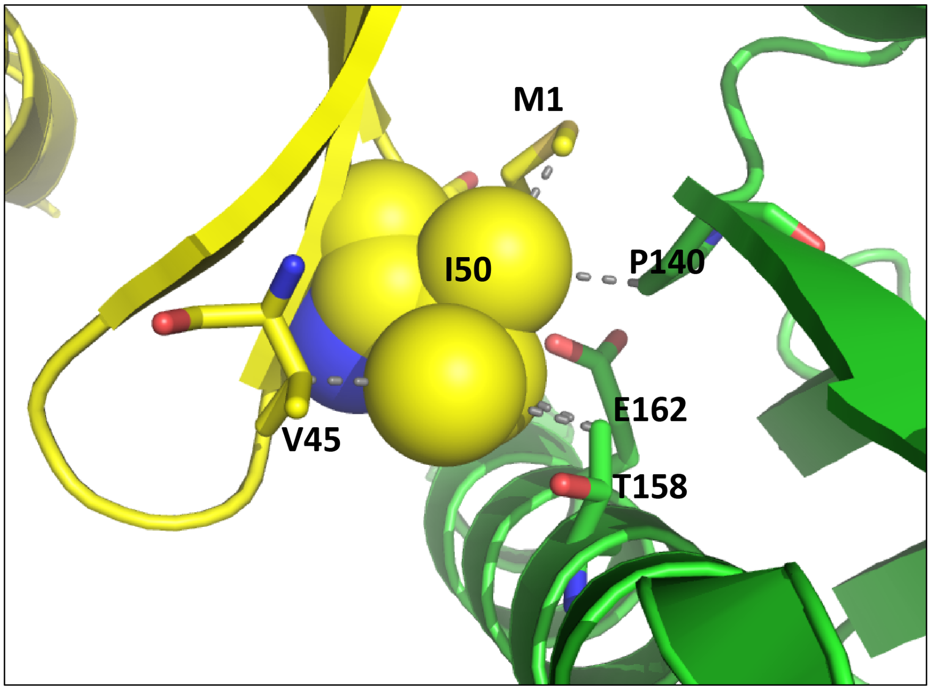

Supplemental Figure 4. Structural modeling indicates that E162L could cooperate with T50I to enhance asymmetric interactions between individual K-Ras molecules.

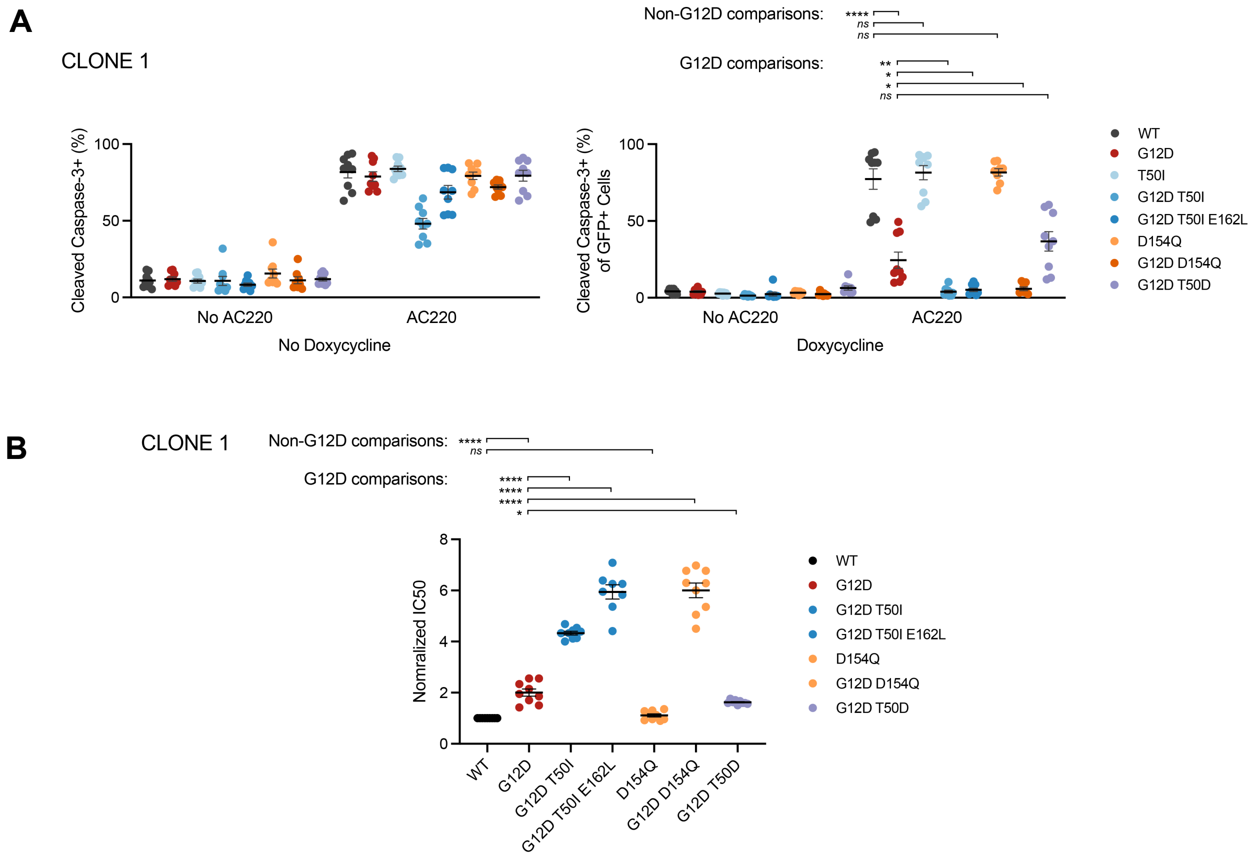

Supplemental Figure 5. Biologic effects of expressing different eGFP-K-Ras fusion proteins in MOLM-13 *KRAS*^{KO} clone 1 cells. (A) Exposure to AC220 (10 nM) for 48 hours induces high levels of apoptosis in clone 1 cells in the absence of dox as assessed by cleaved caspase 3 (CC3) staining. Co-treatment with 2 µg/mL of dox to induce eGFP-K-Ras expression promotes cell survival in an allele-specific manner. Data aggregated from three independent experiments each performed in technical triplicate. (B) CTG assays were performed to assess the viability of clone 1 cells expressing individual eGFP-K-Ras proteins that were cultured in the presence of AC220 (10 nM) and 2 µg/mL of dox for 72 hours. The concentrations of AC220 required to reduce cell growth by 50% (IC50) values were calculated from triplicate samples analyzed in three independent experiments (mean ± SEM). Multiple t-tests were performed using the Holm-Sidak method to correct for multiple comparisons. Adjusted p-value significance is denoted by the number of asterisks: **** < 0.0001, *** ≥ 0.0001 and < 0.001, ** ≥ 0.001 and < 0.01, * ≥ 0.01 and < 0.05, ns ≥ 0.05.

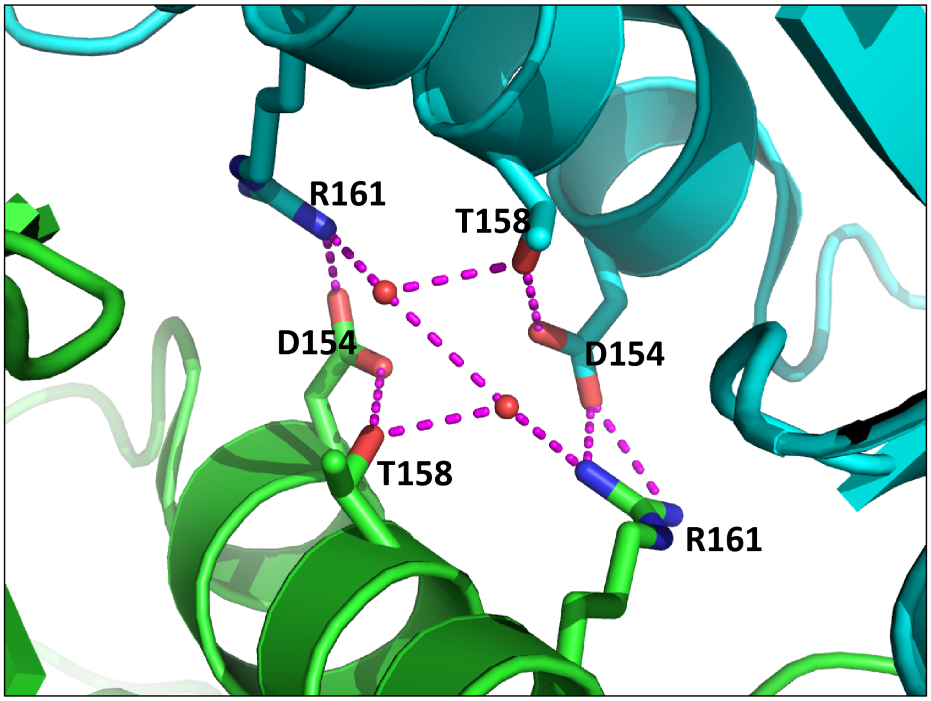

Supplemental Figure 6. Structural modeling of D154Q showing proposed inhibition of symmetric dimer formation (PDB: 5US4).

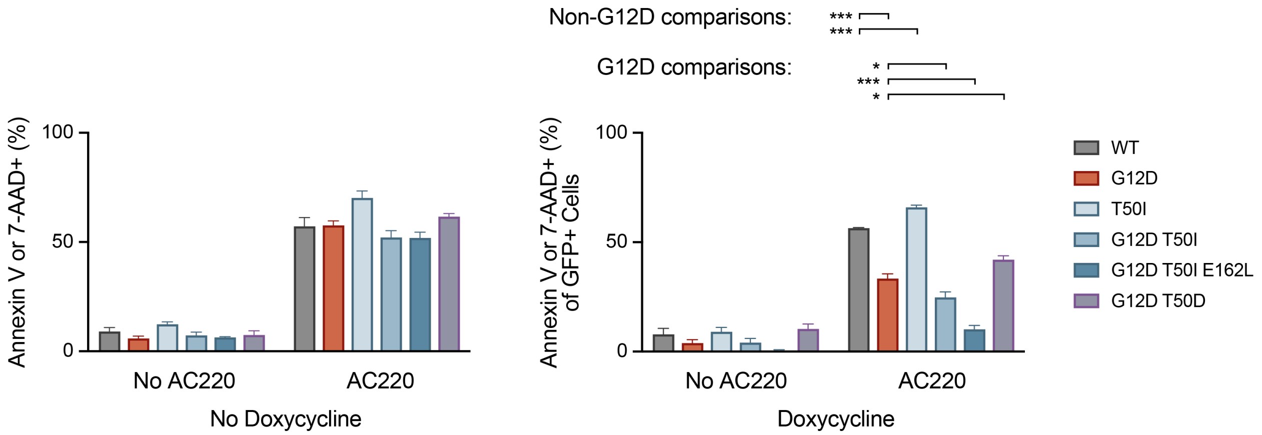

Supplemental Figure 7. Annexin V and 7-AAD response in different eGFP-K-Ras fusion proteins in MOLM-13 *KRAS*^{KO} clone 24 cells. Exposure to AC220 (10 nM) for 48 hours induces high levels of apoptosis in clone 24 cells in the absence of dox as assessed by Annexin V and 7-AAD staining. Co-treatment with 2 μg/mL of dox to induce eGFP-K-Ras expression promotes cell survival in an allele specific manner. Representative result shown from one of three independent experiments each performed in technical triplicate. Values shown are mean ± SEM. Multiple t-tests were performed using the Holm-Sidak method to correct for multiple comparisons. Adjusted p-value significance is denoted by the number of asterisks: **** < 0.0001, *** ≥ 0.0001 and < 0.001, ** ≥ 0.001 and < 0.01, * ≥ 0.01 and < 0.05, ns ≥ 0.05.

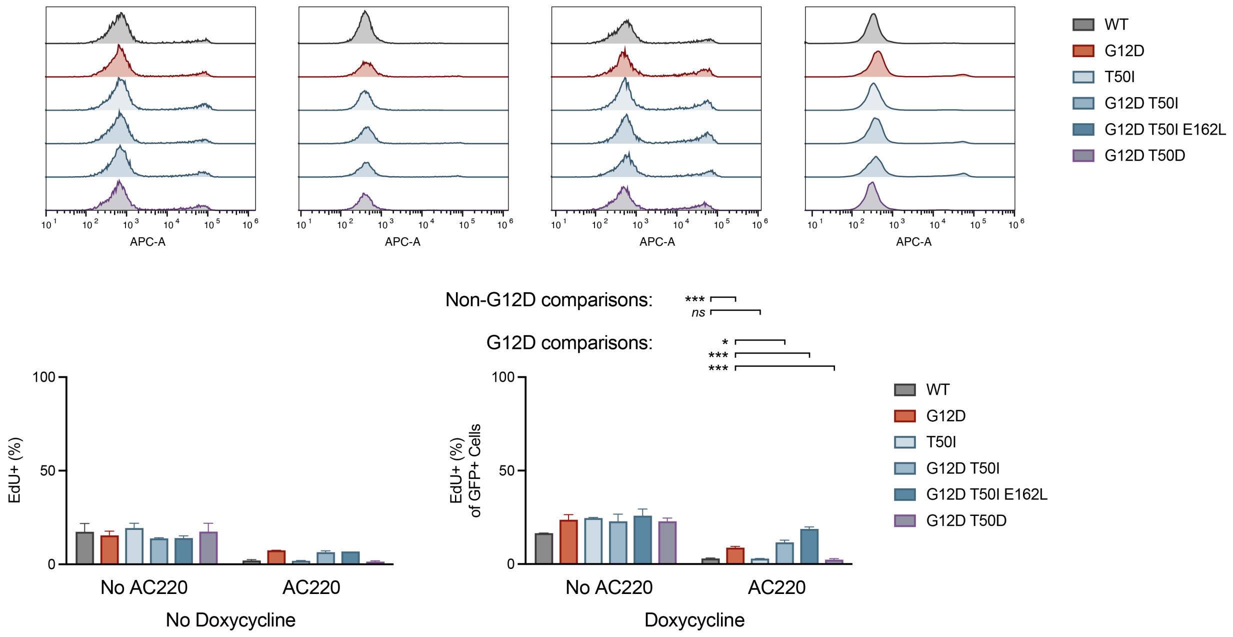

Supplemental Figure 8. EdU uptake in different e-GFP-K-Ras fusion proteins in MOLM-13 *KRAS*^{KO} clone 24 cells. Exposure to AC220 (10 nM) for 24 hours induces cell cycle arrest in clone 24 cells in the absence of dox as assessed by 5-ethynyl-2-deoxyuridine (EdU) uptake. Co-treatment with 2 µg/mL of dox to induce eGFP-K-Ras expression partially restored cell cycle progression in an allele specific manner. Data presented as mean ± SEM from triplicate samples. Multiple t-tests were performed using the Holm-Sidak method to correct for multiple comparisons. Adjusted p-value significance is denoted by the number of asterisks: **** < 0.0001, *** ≥ 0.0001 and < 0.001, ** ≥ 0.001 and < 0.01, * ≥ 0.01 and < 0.05, ns ≥ 0.05.

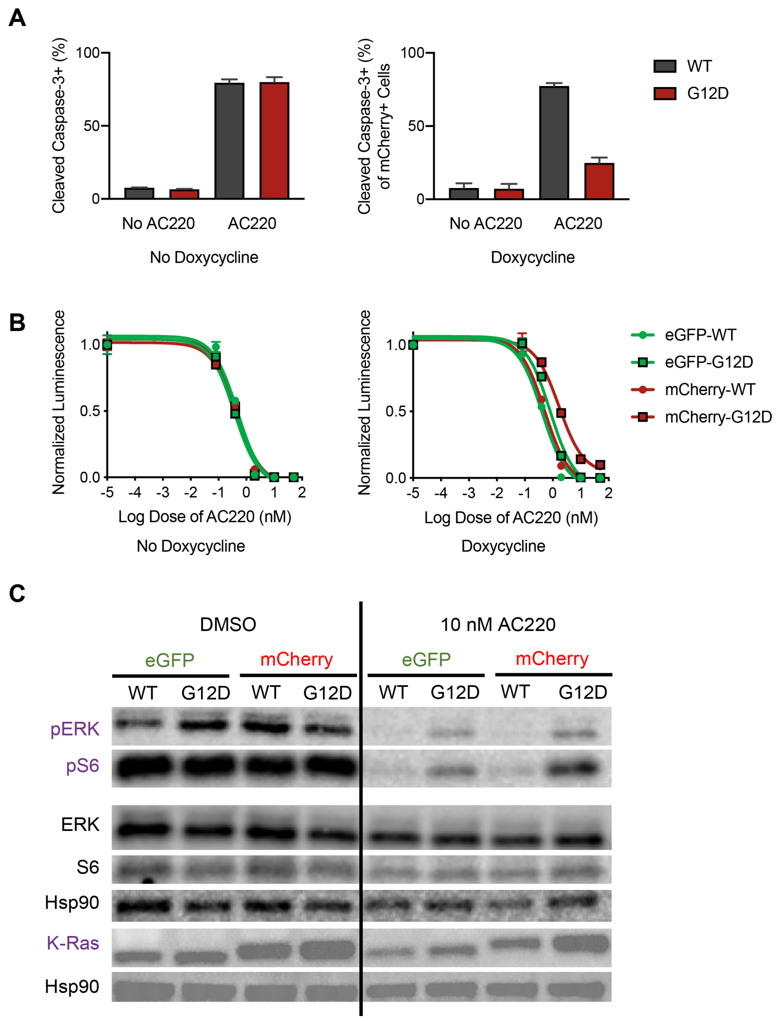

Supplemental Figure 9. Induction of mCherry-K-Ras^{G12D} or eGFP-K-Ras^{G12D} expression suppresses cleaved caspase 3 and promotes AC220 resistance in MOLM-13 *KRAS^{KO}* clone 24 cells in association with persistent ERK and S6 phosphorylation. (A) Cleaved caspase 3 staining of clone 24 cells expressing mCherry-K-Ras^{WT} or mCherry-K-Ras^{G12D} expression (mean ± SEM of triplicate data). The pro-survival activity of mCherry-K-Ras^{G12D} is more potent than that of eGFP-K-Ras^{G12D}. (B) CTG-based dose response analysis showing that mCherry-K-Ras^{G12D} conferred resistance to AC220 more effectively than eGFP-K-Ras^{G12D}. Shown are mean ± SEM of triplicate data. (C) Treatment with 2 μg/mL of dox induces K-Ras expression with AC220 co-treatment suppressing pERK and pS6. Note that clone 24 cells expressing mCherry-K-Ras^{G12D} maintain modestly higher pERK and pS6 levels than clone 24 cells expressing eGFP-K-Ras^{G12D}.

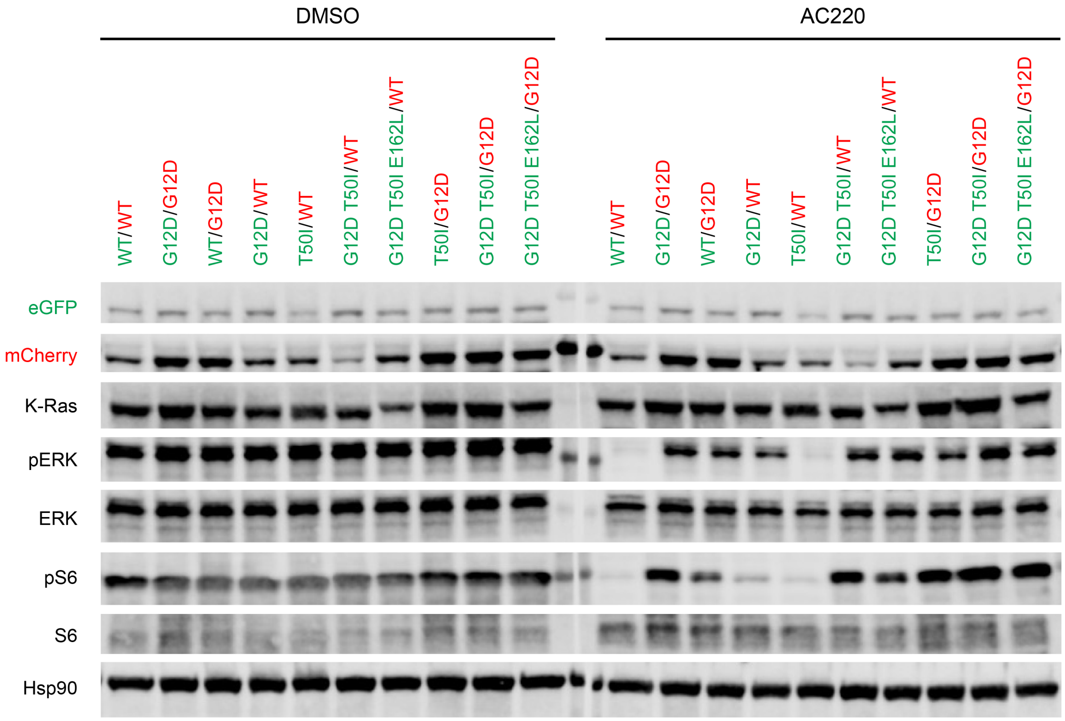

Supplemental Figure 10. Biochemical analysis of MOLM-13 *KRAS*^{KO} clone 24 cells co-expressing eGFP-K-Ras and mCherry-K-Ras proteins showing variable suppression of ERK and S6 phosphorylation. pERK and pS6 levels in cells co-expressing the indicated combinations of eGFP- and mCherry-K-Ras proteins. The reduced K-Ras protein levels seen in cells expressing K-Ras proteins with an E162L substitution are due to failure of the antibody to detect this specific mutation.

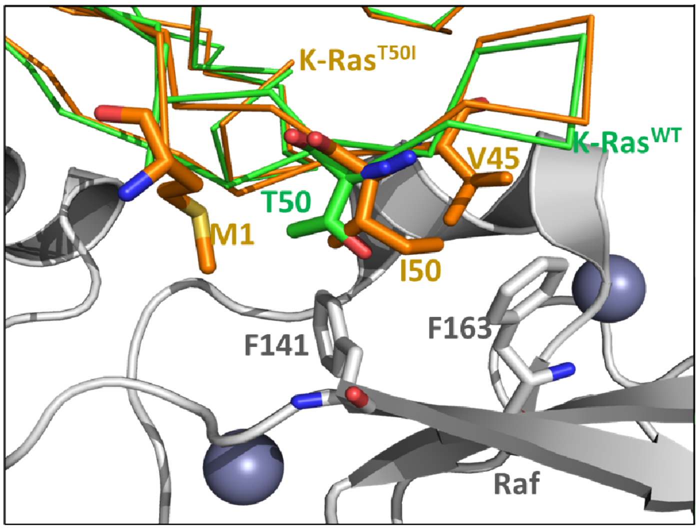

Supplemental Figure 11. Model of Raf-CRD interaction with K-Ras^{T50I}. The co-crystal structure of K-Ras:Raf RBD-CRD (PDB: 6XHB) was aligned with K-Ras^{T50I} to show that T50I mutation could form direct hydrophobic interactions with F141 and F163 of the Raf-CRD.

Table S1. Crystallographic Data and Refinement Statistics

	KRAS^{T50I}-GDP (PDB: 7T1F)
**Data Collection**	
Resolution range (Å)	49.3-2.2
Spacegroup	$P6_122$
Unit cell	a = b = 70.1, c = 337.6
No. of observations	765,079
No. of unique reflections	26,220
Completeness (%)[a]	100.0 (100.0)
R_{sym}(%)[b]	17.4 (133.4)
Redundancy	29.2
**I/Sigma(I)** in the highest resolution bin	3.24
**Refinement statistics**	
Resolution range (Å)	49.3-2.2
No. of reflections: working/free	24,474/1,747
R factor (%)[c]: working/free	21.1/25.0
**Wilson B-factor ($Å^2$)**	29.3
**RMS Deviations**	
Bond length (Å)	0.005
Bond angles (°)	0.861
Ramachandran plot **(% residues)**	
Most favored region	97.1
Additional allowed region	2.9
Disallowed region	0.0

a Numbers in parentheses represent values in the highest resolution shell.

b $R_{sym}=\frac{\sum_h \sum_n |I-\langle I \rangle|}{\sum_h \sum_n \langle I \rangle}$, where I is observed integrated intensity and $\langle I \rangle$ is the averaged integrated intensity taken over n measurements for reflection h.

c R factor$=\frac{\sum_h ||Fo|-|Fc||}{\sum_h |Fo|}$, where F_o is the observed structure factor amplitude and F_c is the calculated structure factor amplitudes based on the refined atomic positions, taken over the h reflections in the observed data set.

Full Unedited Blot for Fig. 1A (part 1 of 3)

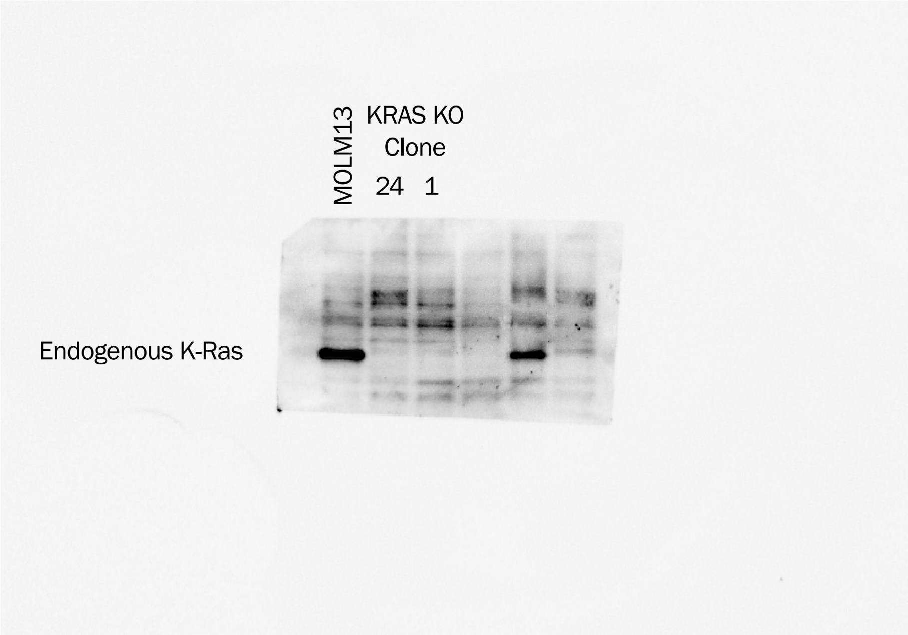

Chen, P-Y, et al. Structural and functional analyses of a germline KRAS mutation provide insights into Raf activation.

Full Unedited Blot for Fig. 1A (part 2 of 3)

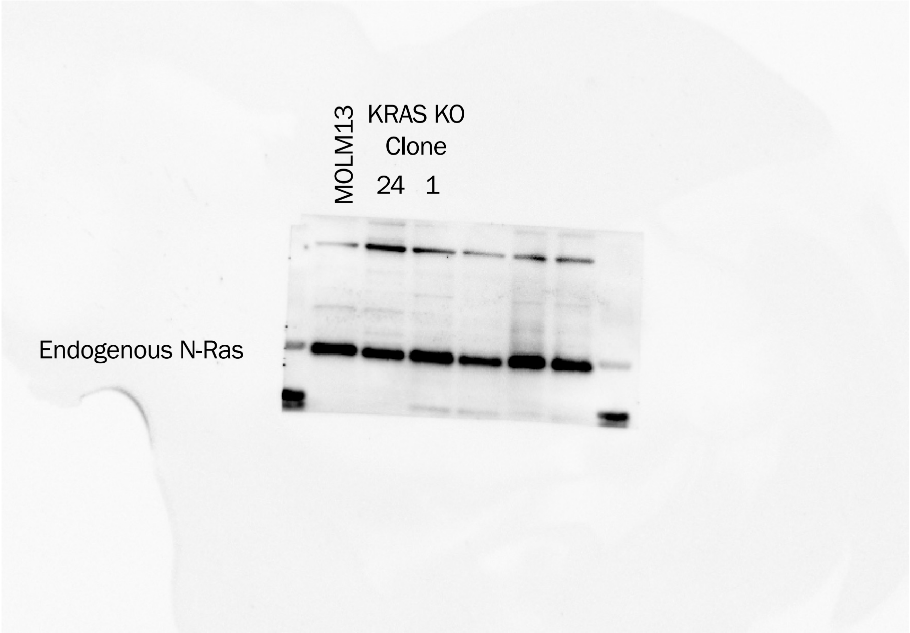

Chen, P-Y, et al. Structural and functional analyses of a germline KRAS mutation provide insights into Raf activation.

Full Unedited Blot for Fig. 1A (part 3 of 3)

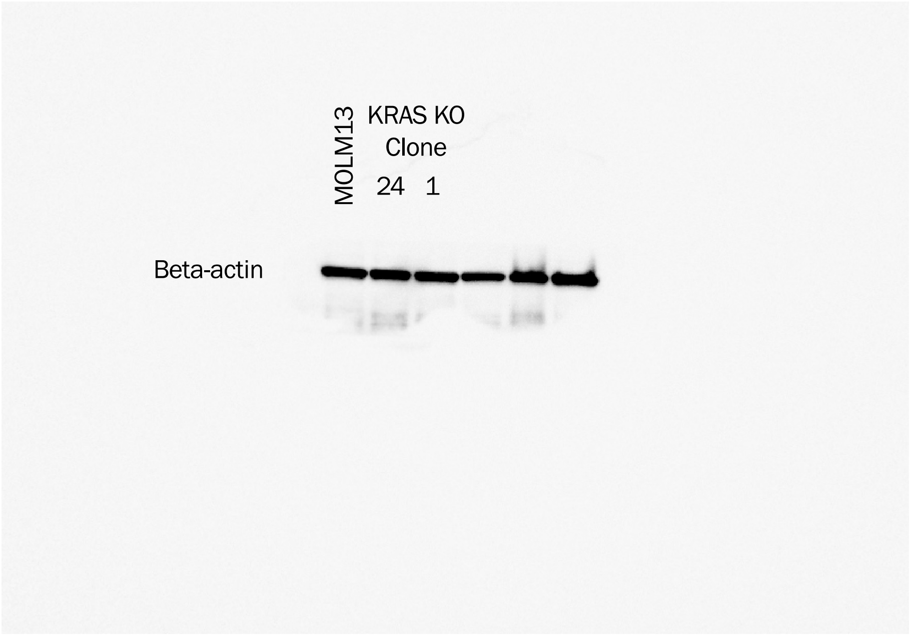

Chen, P-Y, et al. Structural and functional analyses of a germline KRAS mutation provide insights into Raf activation.

Full Unedited Blot for Fig. 1C (part 1 of 2)

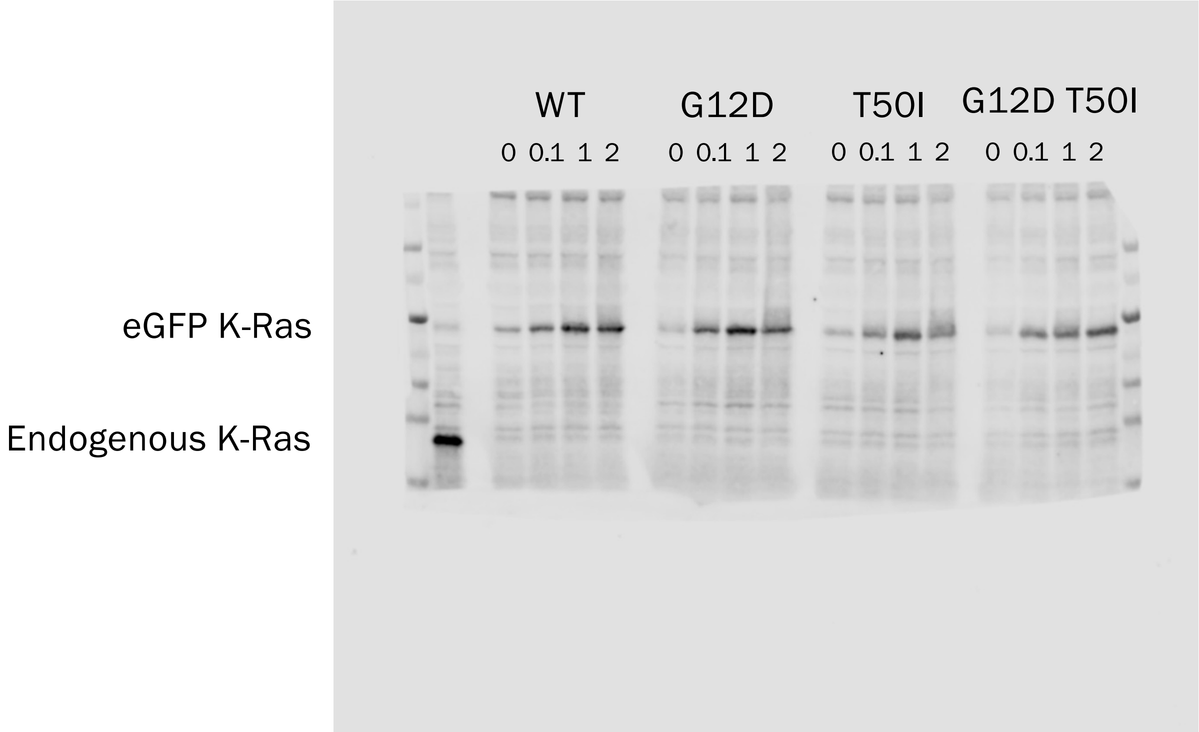

Chen, P-Y, et al. Structural and functional analyses of a germline KRAS mutation provide insights into Raf activation.

Full Unedited Blot for Fig. 1C (part 2 of 2)

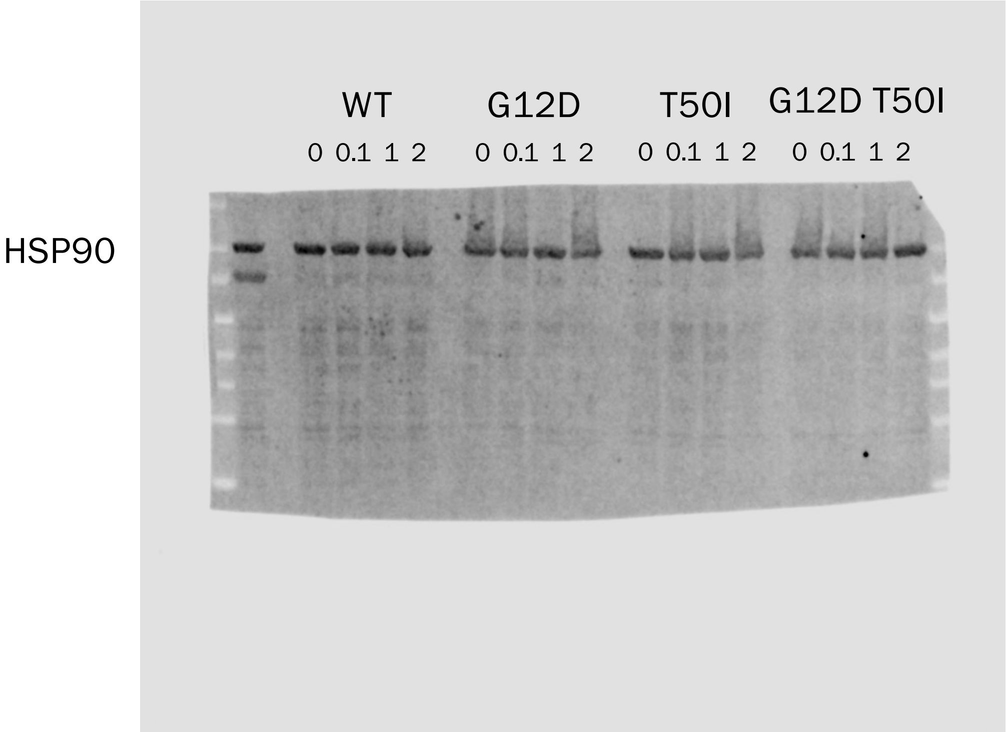

Chen, P-Y, et al. Structural and functional analyses of a germline KRAS mutation provide insights into Raf activation.

Full Unedited Blot for Fig. 3A (part 1 of 3)

Ras-GTP (Pull Down)

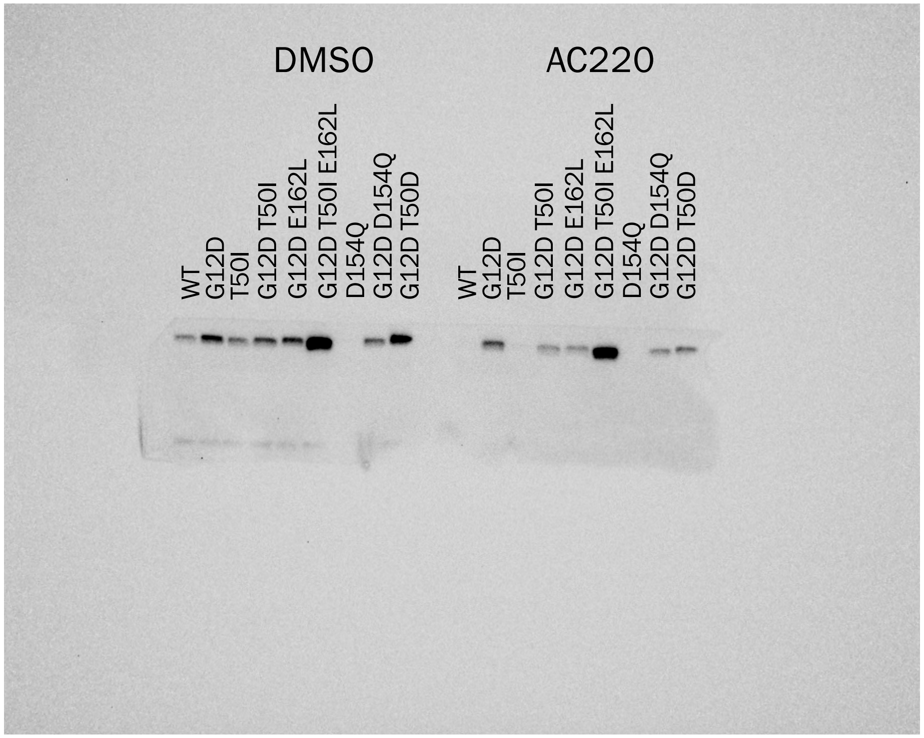

Chen, P-Y, et al. Structural and functional analyses of a germline KRAS mutation provide insights into Raf activation.

Full Unedited Blot for Fig. 3A (part 2 of 3)

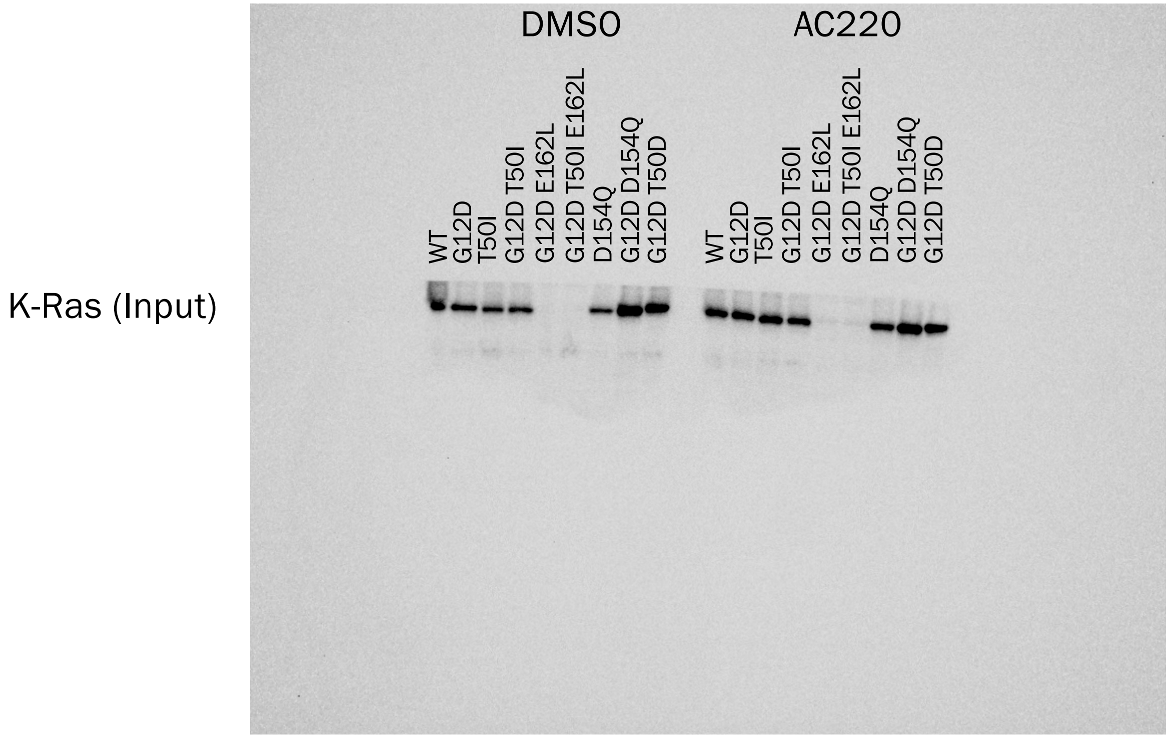

Chen, P-Y, et al. Structural and functional analyses of a germline KRAS mutation provide insights into Raf activation.

Full Unedited Blot for Fig. 3A (part 3 of 3)

eGFP (Input)

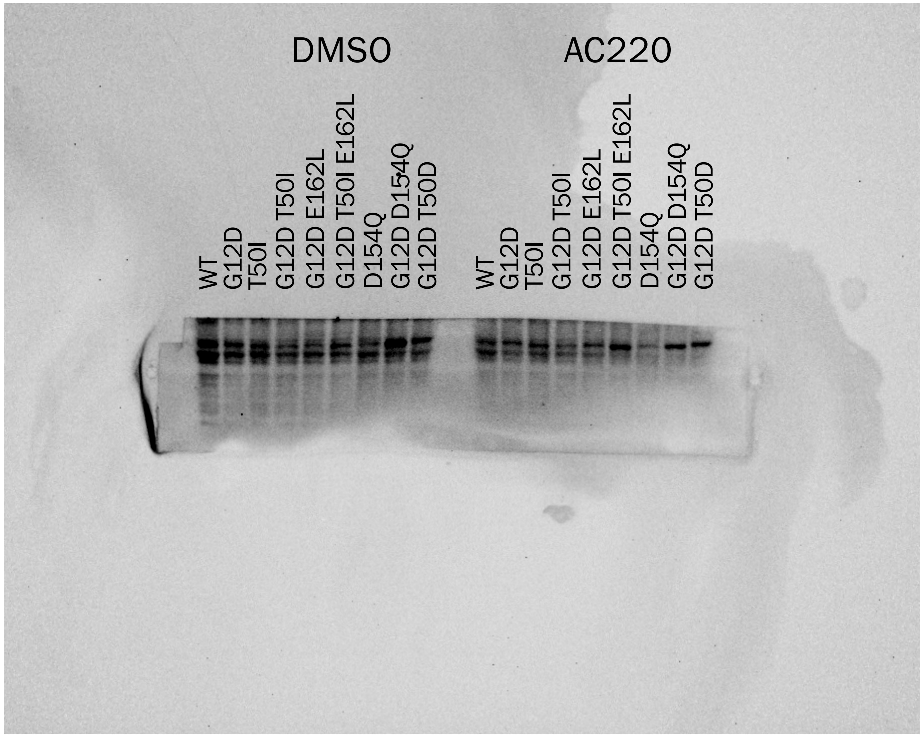

Chen, P-Y, et al. Structural and functional analyses of a germline KRAS mutation provide insights into Raf activation.

Full Unedited Blot for Fig. 3B (part 1 of 5)

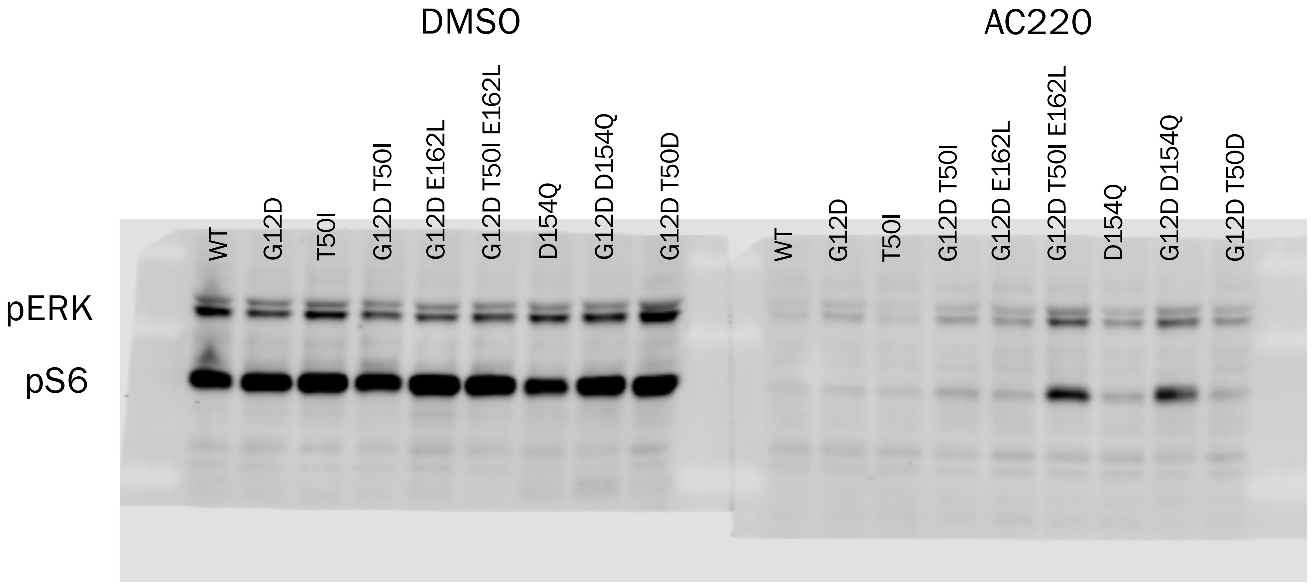

Chen, P-Y, et al. Structural and functional analyses of a germline KRAS mutation provide insights into Raf activation.

Full Unedited Blot for Fig. 3B (part 2 of 5)

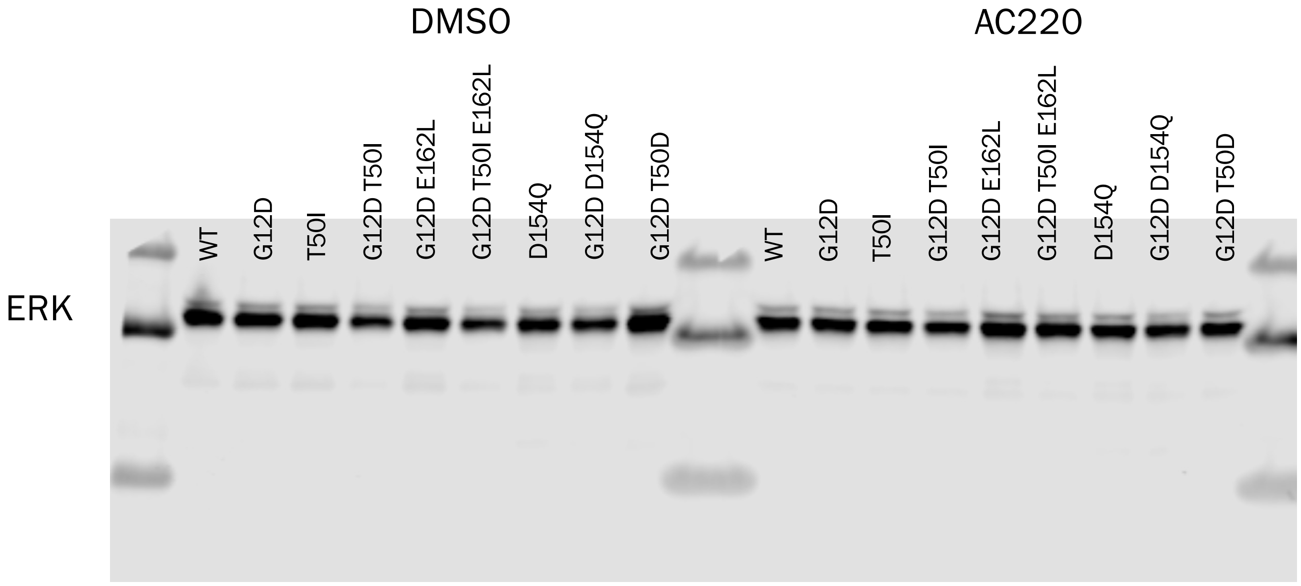

Chen, P-Y, et al. Structural and functional analyses of a germline KRAS mutation provide insights into Raf activation.

Full Unedited Blot for Fig. 3B (part 3 of 5)

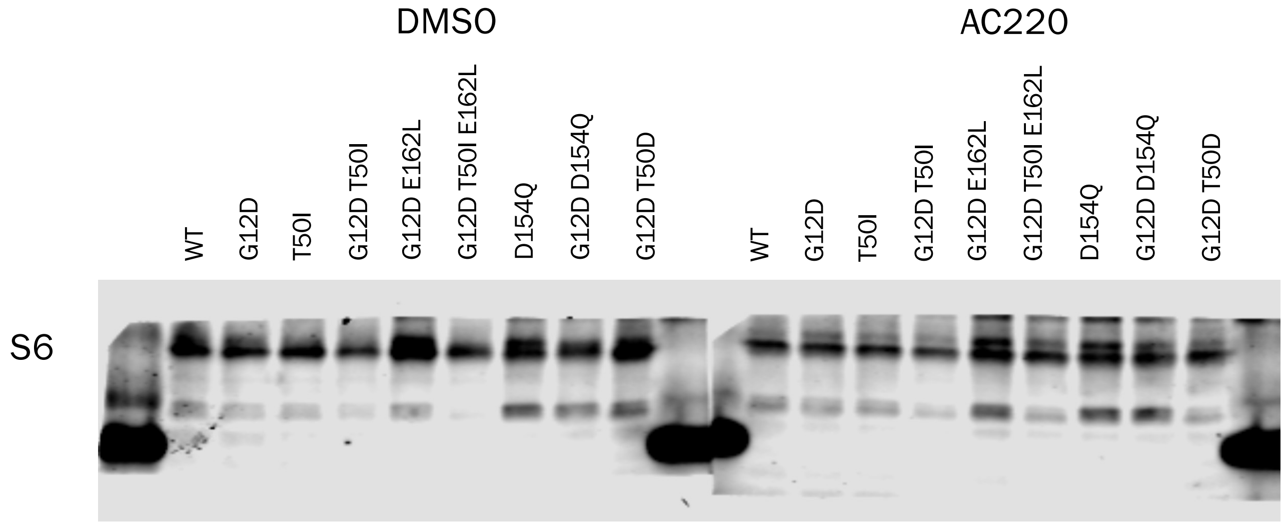

Chen, P-Y, et al. Structural and functional analyses of a germline KRAS mutation provide insights into Raf activation.

Full Unedited Blot for Fig. 3B (part 4 of 5)

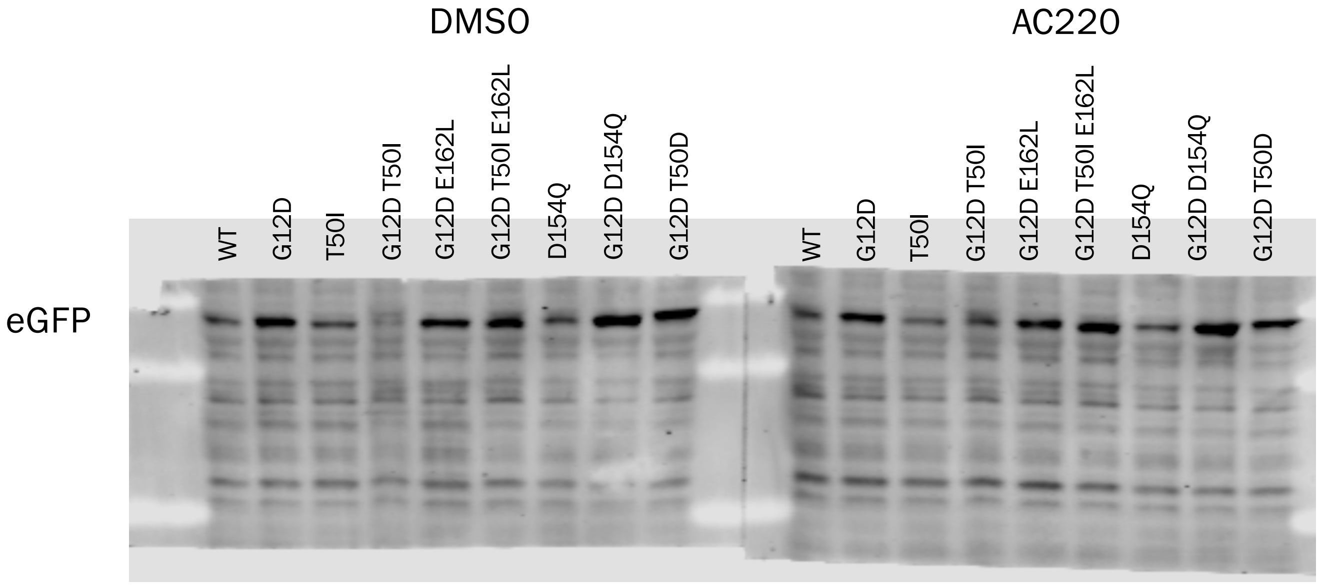

Chen, P-Y, et al. Structural and functional analyses of a germline KRAS mutation provide insights into Raf activation.

Full Unedited Blot for Fig. 3B (part 5 of 5)

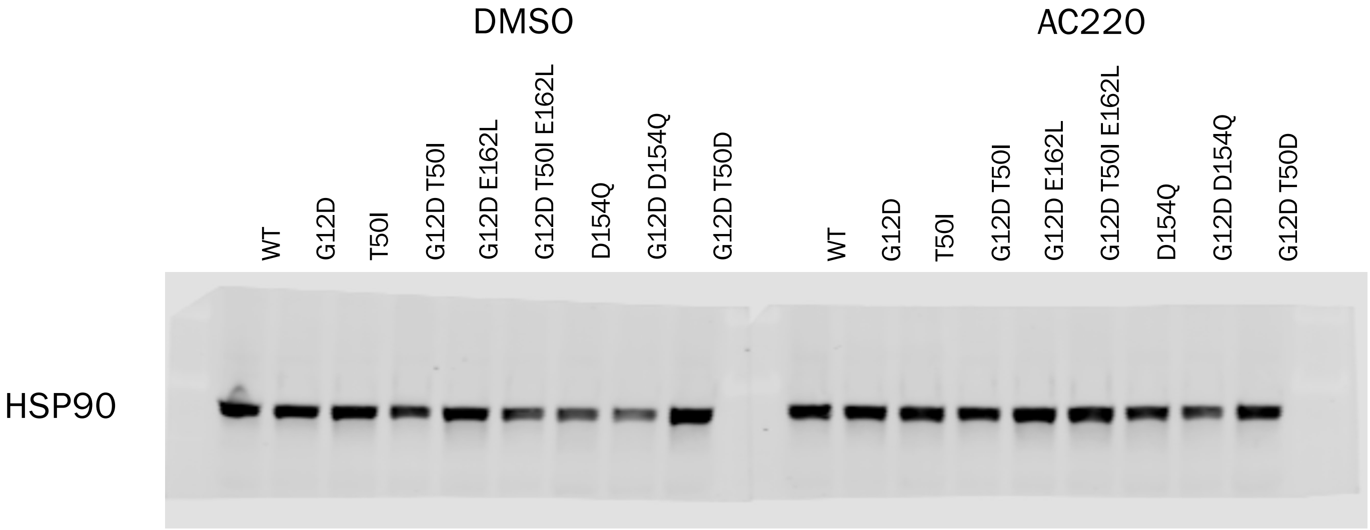

Chen, P-Y, et al. Structural and functional analyses of a germline KRAS mutation provide insights into Raf activation.

Full Unedited Blot for Fig. 3C (part 1 of 7)

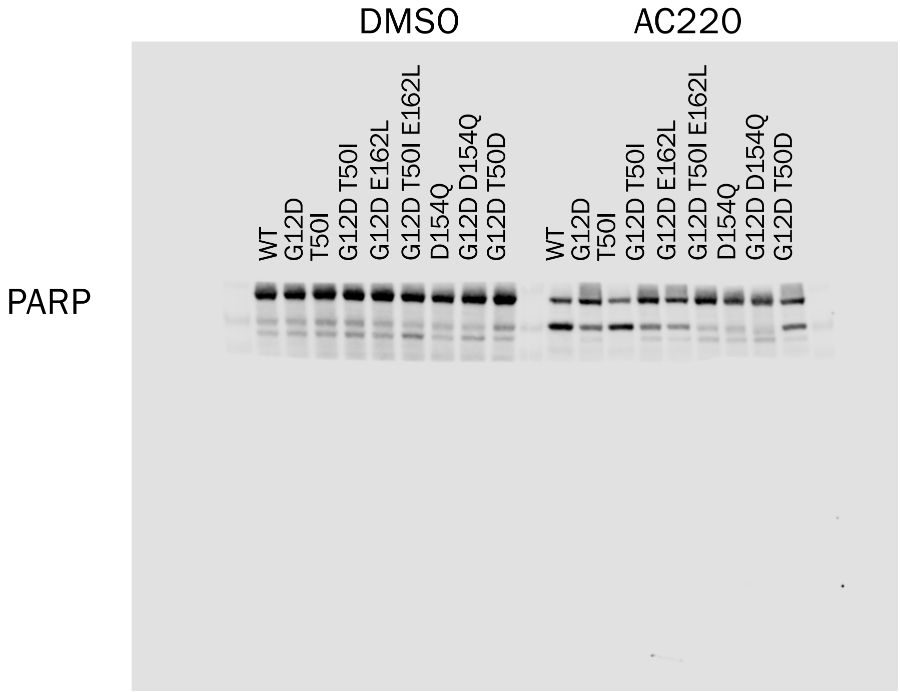

Chen, P-Y, et al. Structural and functional analyses of a germline KRAS mutation provide insights into Raf activation.

Full Unedited Blot for Fig. 3C (part 2 of 7)

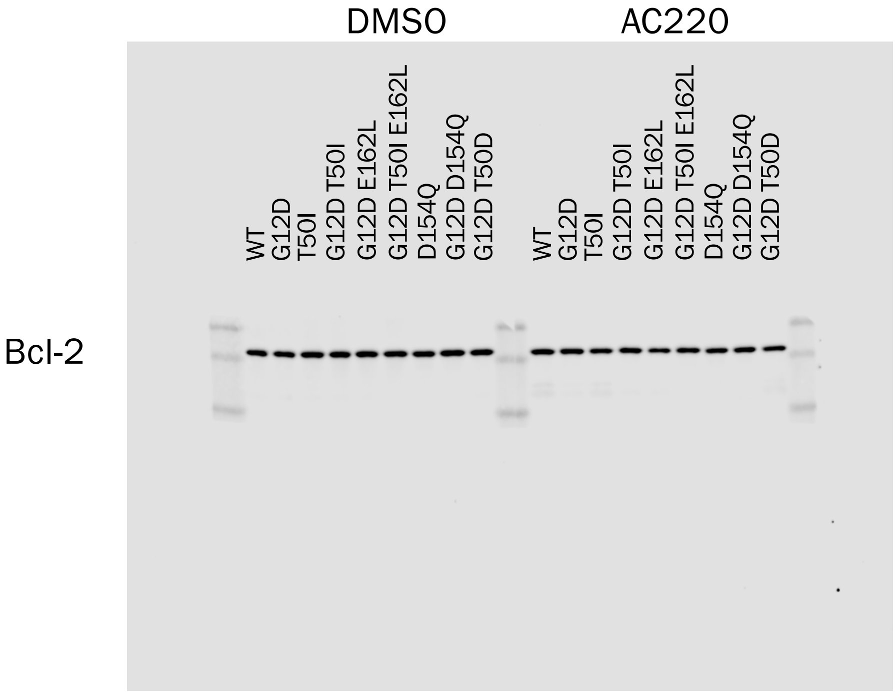

Chen, P-Y, et al. Structural and functional analyses of a germline KRAS mutation provide insights into Raf activation.

Full Unedited Blot for Fig. 3C (part 3 of 7)

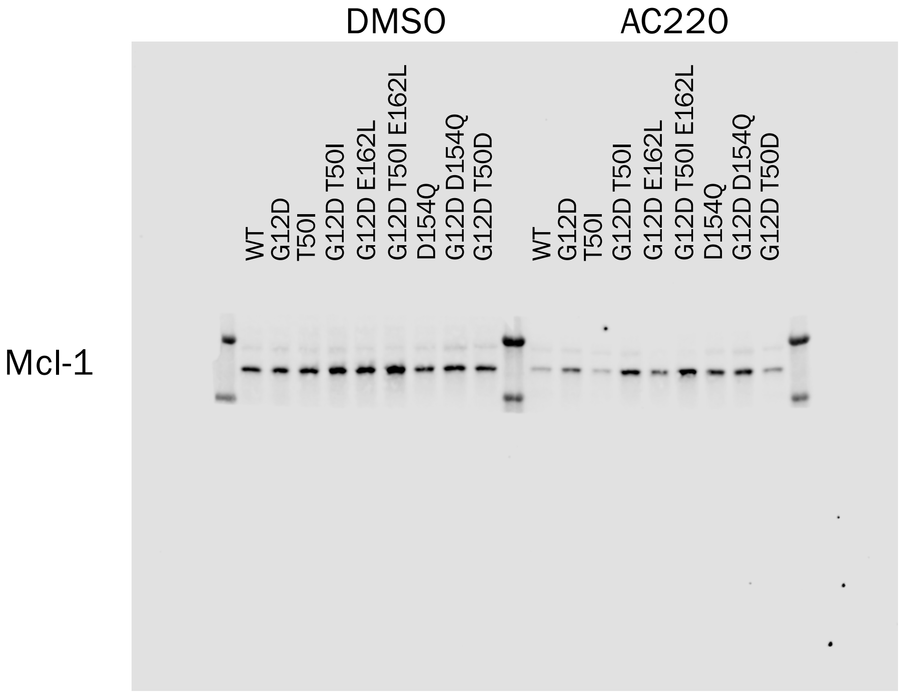

Chen, P-Y, et al. Structural and functional analyses of a germline KRAS mutation provide insights into Raf activation.

Full Unedited Blot for Fig. 3C (part 4 of 7)

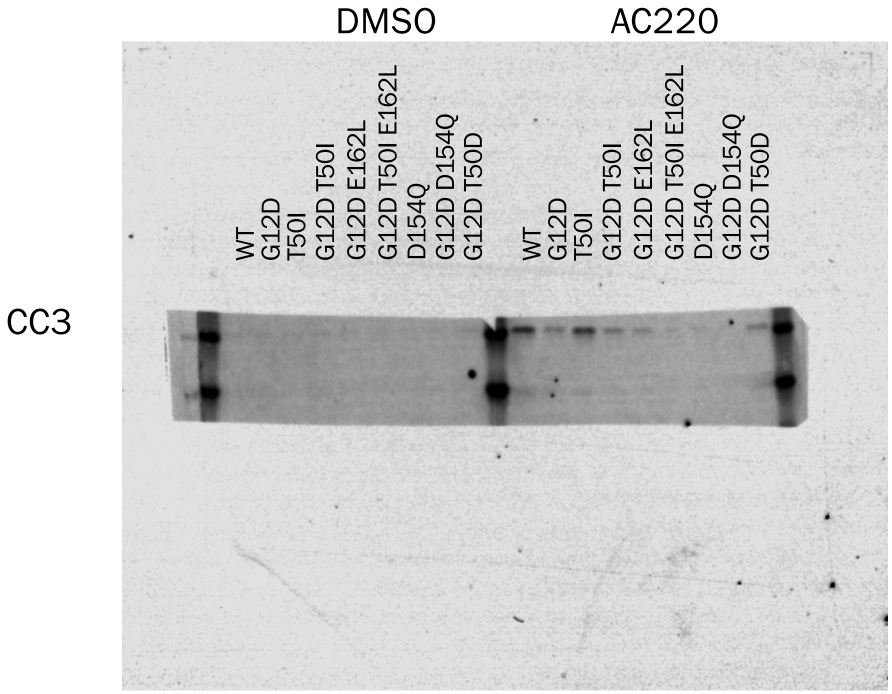

Chen, P-Y, et al. Structural and functional analyses of a germline KRAS mutation provide insights into Raf activation.

Full Unedited Blot for Fig. 3C (part 5 of 7)

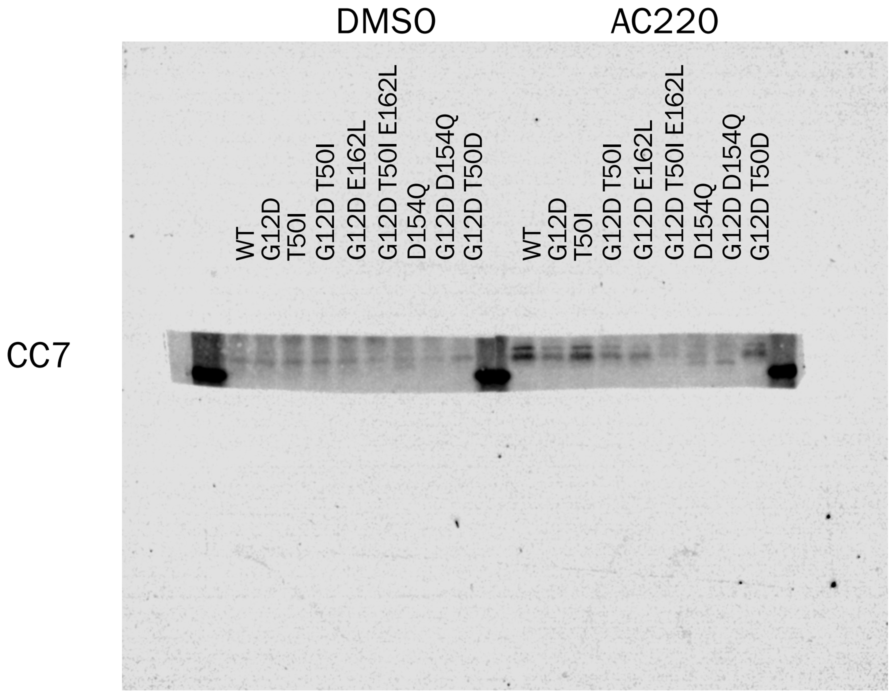

Chen, P-Y, et al. Structural and functional analyses of a germline KRAS mutation provide insights into Raf activation.

Full Unedited Blot for Fig. 3C (part 6 of 7)

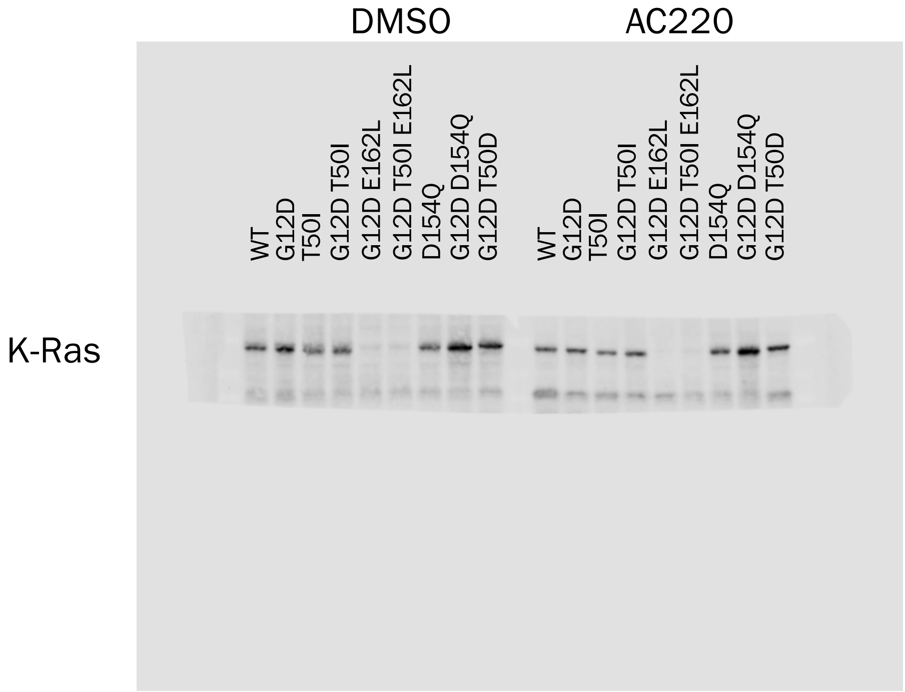

Chen, P-Y, et al. Structural and functional analyses of a germline KRAS mutation provide insights into Raf activation.

Full Unedited Blot for Fig. 3C (part 7 of 7)

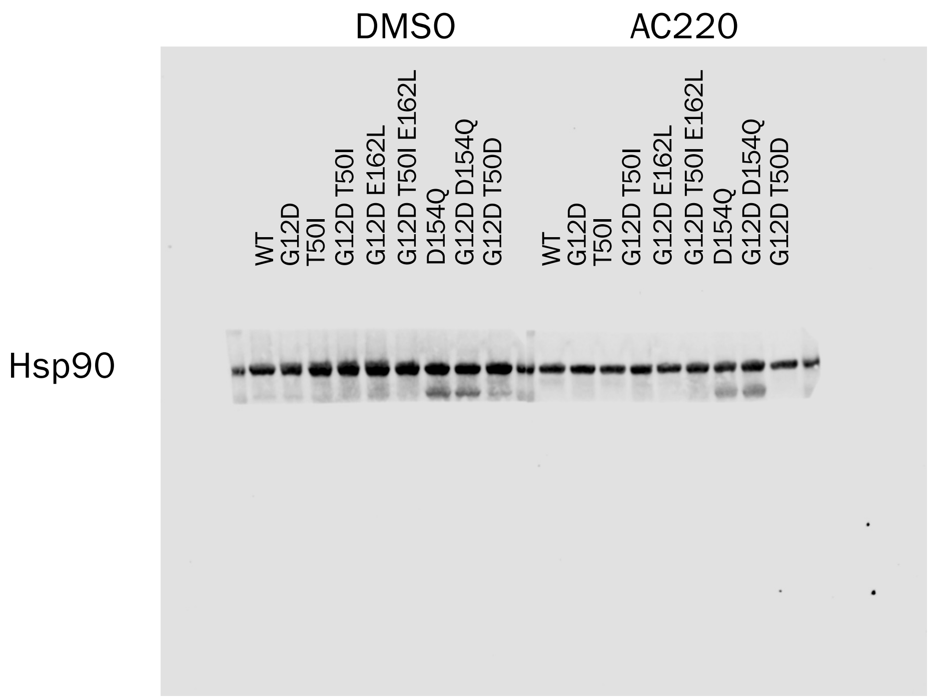

Chen, P-Y, et al. Structural and functional analyses of a germline KRAS mutation provide insights into Raf activation.
